# Global, regional, and national trends and patterns in physical activity research since 1950: a systematic review

**DOI:** 10.1186/s12966-020-01071-x

**Published:** 2021-01-07

**Authors:** Andrea Ramírez Varela, Gloria Isabel Nino Cruz, Pedro Hallal, Cauane Blumenberg, Shana Ginar da Silva, Deborah Salvo, Rafaela Martins, Bruna Gonçalves Cordeiro da Silva, Eugen Resendiz, Maria Catalina del Portillo, Luciana Zaranza Monteiro, Selina Khoo, Kar Hau Chong, Marcelo Cozzensa da Silva, Alice Mannocci, Ding Ding, Michael Pratt

**Affiliations:** 1 School of Medicine, Universidad de los Andes, Cra 7 #116-05, 11001000 Bogotá, Colombia; 2 School of Physiotherapy, Universidad Industrial de Santander, Bucaramanga, Colombia; 3 Post-Graduate Program in Epidemiology, Federal University of Pelotas, Pelotas, Brazil; 4 Post-Graduate in Biomedical Science, School of Medicine, Federal University of Fronteira Sul, Chapecó, Brazil; 5 Prevention Research Center, Brown School, Washington University in St. Louis, St. Louis, USA; 6 University Center of the Federal District, Brasília, Brazil; 7 University of Malaya, Kuala Lumpur, Malaysia; 8 Mercatorum University, Rome, Italy; 9 University of Sydney, Sydney, Australia; 10 University of California San Diego Herbert Wertheim School of Public Health and Longevity Science, San Diego, USA

**Keywords:** Physical activity, Research, Epidemiology, Public health, Surveillance

## Abstract

**Background:**

National, regional and global scientific production and research capacity for physical activity - PA may contribute to improving public health PA policies and programs. There is an uneven distribution of research productivity by region and country income group, where countries with the highest burden of non-communicable diseases attributable to physical inactivity having low research productivity. A first step towards improving global research capacity is to objectively quantify patterns, trends, and gaps in PA research. This study describes national, regional and global trends and patterns of PA research from 1950 to 2019.

**Methods:**

A systematic review using searches in PubMed, SCOPUS and ISI Web of Knowledge databases was conducted in August 2017 and updated between January and May 2020. The review was registered at the PROSPERO database number CRD42017070153. PA publications per 100,000 inhabitants per country was the main variable of interest. Descriptive and time-trend analyses were conducted in STATA version 16.0.

**Results:**

The search retrieved 555,468 articles of which 75,756 were duplicates, leaving 479,712 eligible articles. After reviewing inclusion and exclusion criteria, 23,860 were eligible for data extraction. Eighty-one percent of countries (*n* = 176) had at least one PA publication. The overall worldwide publication rate in the PA field was 0.46 articles per 100,000 inhabitants. Europe had the highest rate (1.44 articles per 100,000 inhabitants) and South East Asia had the lowest (0.04 articles per 100,000 inhabitants). A more than a 50-fold difference in publications per 100,000 inhabitants was identified between high and low-income countries. The least productive and poorest regions have rates resembling previous decades of the most productive and the richest.

**Conclusion:**

This study showed an increasing number of publications over the last 60 years with a growing number of disciplines and research methods over time. However, striking inequities were revealed and the knowledge gap across geographic regions and by country income groups was substantial over time. The need for regular global surveillance of PA research, particularly in countries with the largest data gaps is clear. A focus on the public health impact and global equity of research will be an important contribution to making the world more active.

**Supplementary Information:**

The online version contains supplementary material available at 10.1186/s12966-020-01071-x.

## Introduction

Scientific evidence on the multiple health benefits of regular physical activity across the lifespan has been documented over the last five decades [1–4]. Despite such mounting evidence, in 2016 the World Health Organization (WHO) estimated that the global prevalence of physical inactivity among adults was 27.5%. Overall, this prevalence has ranged between 23 and 32% during the last two decades with some variations. This is primarily a result of differences in measurement methods and/or cut-points for meeting recommendations, rather than significantly change in population prevalence over time [5]. The fact that one in four adults in the world do not meet physical activity recommendations makes physical inactivity one of the leading underlying causes and modifiable behavioral factors of preventable global morbidity and mortality [5].

Improving national, regional, and global scientific production and research capacity has been identified as a critical strategy for improving public health policies and programs for physical activity [6–9]. However, The 2012 and 2016 Lancet Physical Activity Series as well as The 2015 Global Observatory for Physical Activity – GoPA! [10] Country Cards and Almanac [10] documented unequal distributions of research productivity by region and country income level. Countries with the highest burden of preventable non-communicable diseases attributable to physical inactivity [11, 12] tended to have lower research productivity. A first step towards improving global research capacity is to better understand the trends in research publications in the field. To date, there have been few analyses objectively documenting the trends and patterns of global physical activity research [12]. The aim of this study was to describe national, regional and global trends and patterns of physical activity research from 1950 to 2019. Specifically, we identified themes of physical activity research by classifying publications into the following topics: 1) physical activity levels, trends and measurement, 2) determinants of physical activity, 3) health consequences of physical activity, 4) physical activity interventions, and 5) physical activity policy and practice.

## Methods

### Study design

A systematic review to estimate country-specific physical activity-related research was conducted in August 2017 and updated in January 2020. The databases used included PubMed, SCOPUS and ISI Web of Knowledge. Data extraction and review took place between August 2017 and November 2018 for the first search and between January and May 2020 for the update. This systematic review followed PRISMA guidelines [13] and was registered at the PROSPERO database with the number CRD42017070153 [14]. EndnoteX8 was used to manage country-specific reference libraries. This study was approved by the Research Ethics Committee of the Faculty of Physical Education (n° 522.064) at the Federal University of Pelotas, Brazil. CAAE n° 67,102,116.0.0000.5313.

### Identification and classification of countries and country-specific general information

Starting with the World Bank [15] list of 215 countries [16], we divided the United Kingdom into England, Scotland, Wales and Northern Ireland, and we combined information from China and Taiwan as the Greater China Area. Our final list comprised 217 countries. Countries were grouped by region, following the World Health Organization [17] 2019 regional classification [18] (EURO - Europe; AFRO - Africa; PAHO - The Americas and the Caribbean; EMRO - Eastern Mediterranean; WPRO - Western Pacific; SEARO - South-East Asia) and country income level following the 2019 World Bank classification (HICs - high income countries, UMICs - upper middle income countries; LMICs - lower middle income countries; and LICs - low income countries) [16]. Population estimates by year were obtained from the World Bank [16] and national statistics sources in the case of England, Northern Ireland, Scotland and Wales [19–22].

### Search terms and strategies

Search terms for “physical activity” (in title or abstract) and country name in English (anywhere in the title, abstract, text or affiliation) were used. ‘Physical activity’ terms included both those referring to physical movement, as well as those encompassing the concept of sedentary behaviours (sitting across all domains, including occupation, leisure, domestic and travel) different than TV viewing only. The ‘physical activity’ search terms used were as follows: physical activity OR physically active OR physical inactivity OR physically inactive OR fitness OR exercise* OR walk OR walking OR sedentary OR active transport* OR active transit OR active travel OR commute* OR active commuting OR bicycle OR bicycling OR bike OR biking OR active living OR active-living. All titles and abstracts identified in the search were screened by pairs of authors applying pre-established inclusion and exclusion criteria to select studies for a complete reading. Extraction was conducted by pairs of authors and in case of doubts regarding classification, a third author was consulted. References were managed and imported to Endnote X8 (see appendix supplementary appendix for a full list of search terms).

### Inclusion and exclusion criteria

In this study the country was the main unit of analysis. Publications included in this study were those described in the titles and abstracts as physical activity studies, either observational studies or experimental/intervention studies (includes quasi-experimental). Reviews, meta-analyses, case reports, editorials, commentaries, conference abstracts, national plans, surveillance papers, discussions or letters to the editor were included if they appeared in the search and if the article included data on that specific country. The Lancet Series and multi country articles were counted as part of specific country’s productivity if local data were included. Therefore, a publication could be counted towards two or more countries’s productivity if local data was included. Studies on exercise physiology, on athletes or military populations were excluded. Dates of publication were restricted to 01/01/1950–31/12/2019. There were no age or study design restrictions. Articles written in English, Spanish and Portuguese were included. We also included publications written in another language, but with an English abstract which contained sufficient data required for extraction.

### Characteristics of physical activity studies

1. *Study design:* (a) observational, (b) experimental/intervention. If observational study: (a) cross-sectional, (b) longitudinal, (c) case-control.
2. *Age group:* (a) general adult population > =18 years < 60 years, (b) children and adolescents < 18 years, (c) specific for older adult population > =60 years, (d) more than one age group; The age cut-points listed here were used as a rough guide and the specific age definition may differ by studies.
3. *Physical activity study type:* Studies were organized into one of the following five categories as previously described [12]: (a) physical activity levels, trends and measurement, (b) determinants of physical activity, (c) health consequences of physical activity, (d) physical activity interventions, and (e) physical activity policy and practice;
4. *Study topic related to physical activity promotion (according to the Bangkok Declaration and the WHO SDGs report* [23, 24]*)*: (a) cardiovascular disease (e.g. hypertension, hypercholesterolemia, metabolic syndrome, diabetes); (b) cancer; (c) mental health and illness (e.g cognition, memory, attention, dementia, depression); (d) earth/environmental/atmospheric sciences (e.g. climate change, global warming); (e) Built and natural environment (Built and green spaces); (f) sedentary time; (g) population with disabilities (disability is an impairment that may be cognitive, developmental, intellectual, mental, physical, sensory, neurological, or some combination of these. ex: cerebral palsy and paraplegia, quadriplegia); (h) nutrition (e.g. obesity, BMI indices, nutrition assessment); (i) methods (e.g validation and measurement studies); (j) International policy documents and recommendations (e.g WHO Millennium and/or Sustainable development goals documents, Global Action Plan for Physical Activity –GAPPA, Bangkok declaration [17, 25]); (k) healthy lifestyle studies (physical activity within the context of other lifestyle risk factors), or (l) other;
5. *Study included objective measurement of physical activity:* yes or no
6. *Study included multiple countries:* yes or no

### Country-level physical activity research indicators

1. *Total number of articles per country from 1950 to 2019:* Total publications per country, resulting from the final selection of articles for the systematic review.
2. *Contribution of the country to physical activity research worldwide from 1950 to 2019:* Estimated as the percentage of publications per country (total articles per country/ total of articles worldwide) *100.
3. *Physical activity productivity indicator from 1950 to 2019 (main dependent variable):* Total number of publications on physical activity per 100,000 inhabitants. Estimated for each country, world region and globally as an overall rate including all data from 1950 to 2019, and by decades 60’s, 70’s, 80’s, 90’s, 2000’s, 2010’s [26].
  - For example, the total number of articles in the 60’s decade was calculated as the sum of articles found each year during 1960–1969. The mean population in the 60’s was calculated as the sum of the population found each year from (1960–1969)/10.

### Statistical analyses

Descriptive statistics were estimated. Trend analysis, Chi-square test and Fisher-Hayter pairwise comparisons test were conducted with physical activity productivity indicator by decade by World Bank income categories. Statistical analyses were performed in STATA version 16.0 software (Stata Corp. College Station, TX, USA).

## Results

**Fig. 1:**
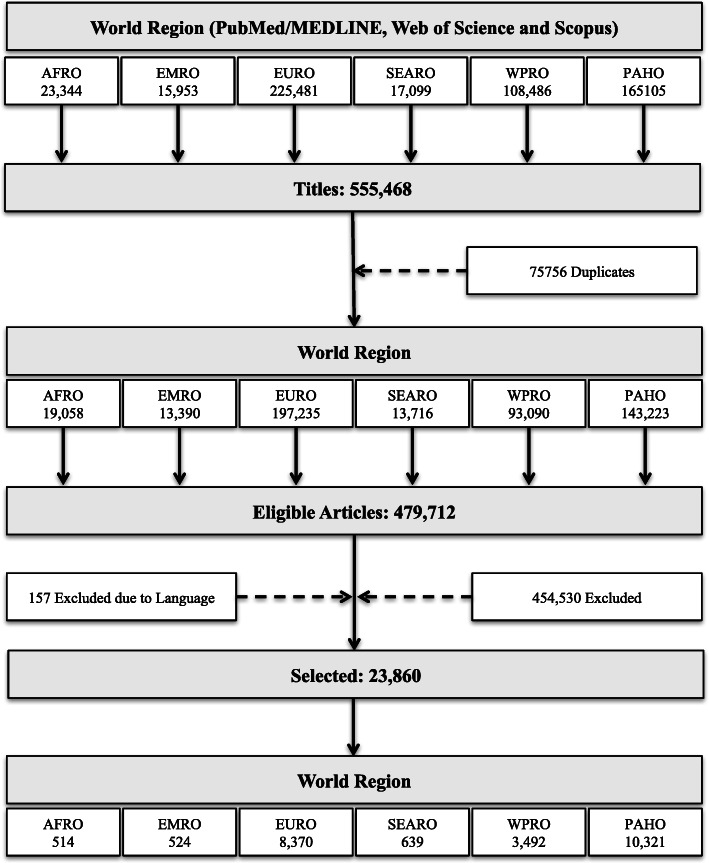
Systematic review flowchart

This systematic review collected information describing global, regional, and national trends and patterns of physical activity research from 1950 to 2019. The automated search strategy retrieved 555,468 articles of which 75,756 were duplicates, leaving 479,712 eligible articles. After reviewing inclusion and exclusion criteria 23,860 were selected for data extraction. Figure 1 shows the systematic review flowchart.

### Where does physical activity research take place?

Of the 217 countries in the world, 176 (81.1%) had at least one physical activity publication between 1950 and 2019 (Table 1). The number of publications ranged from 1 to 6111 articles with a median of 13 articles. PAHO (48.4%) and EURO (34.8%) accounted for 83.2% of total global publications, WPRO 15%, AFRO, EMRO and SEARO approximately 2% each (Table 1). Most of the countries showed a marked increase in the number of publications in the last two decades (2000–2019) when compared to previous decades (1950–1990).

**Table 1:** Physical activity research estimates by world region and income group

	Worldcountries *(***n*** = 217)	Countries withpublications(***n*** = 176)	Number of articles meeting theinclusion criteria (***n*** = 23,860)	Mean publication rate per100,000 inhabitants**	Contribution tothe total (%)
World region ***					
Africa	46	32	514	0.09	2.2
Eastern Mediterranean	23	21	524	0.13	2.2
Europe	62	51	8372	1.44	35.1
The Americas and the Caribbean	44	34	10,321	1.43	43.3
South East Asia	11	10	639	0.04	2.7
Western Pacific	31	28	3490	0.23	14.6
Income group ****+					
High income	81	65	19,144	1.88	80.2
Upper middle income	55	47	3534	1.16	14.8
Lower middle income	46	42	915	0.05	3.8
Low income	34	22	267	0.06	1.1

* Database search was conducted for the 217 world countries GoPA! list. Population, world region and income group classifications according to the World Bank. United Kingdom was divided in its 4 countries (England, Scotland, Wales and Northern Ireland)

**Regional and income group population was estimated as the sum of population average by decades from 1960 to 2019

+ Fisher-Hayter pairwise comparisons test and Chi square test for trend of publication rate by income level *p* < 0.005

***World Health Organization regions: Africa - AFRO; Eastern Mediterranean EMRO; Europe - EURO; The Americas and the Caribbean - PAHO; South East Asia - SEARO; Western Pacific - WPRO

****World Bank country income classification: High Income - HICs; Upper middle income - UMICs; Lower middle income - LMICs; Low income - LICs

High income countries produced 80% of the publications, upper middle income countries 15%, lower middle income countries 4% and low income countries 1% (Table 2). Productivity was less concentrated in the lower and middle income country categories. An inverse pattern was observed where the five most productive countries in the low income group accounted for more than 75% of the group’s publications, compared with 70% for the middle income group and 56% in the high income group. A more than 50-fold difference in publications was observed between the five most productive high income countries compared to the five most productive low income countries.

**Table 2:** Average global share of physical activity research publications, 1950–2016

Total number of physical activity publications (***n*** = 23.860)			
Income group***	Total publicationsper group	Top 5 countries contributionto the total in %	Group contributionto the total in %
**High income (65 countries)**	19,132		
Top 5 countries	10,851		
Top 5/Total High income (%)	56.72	45.5	80.2
**Upper middle income (47 countries)**	3546		
Top 5	2549		
Top 5/Total UMI (%)	71.88	10.7	14.9
**Lower middle income (42 countries)**	914		
Top 5	629		
Top 5/Total LMI (%)	68.82	2.6	3.8
**Low income (22 countries)**	268		
Top 5	204		
Top 5/Total LI (%)	76.12	0.9	1.1

****World Bank country income classification: High Income - HICs; Upper middle income - UMICs; Lower middle income - LMICs; Low income – LICs

### Physical activity research publication rates

The overall worldwide publication rate for the period from 1950 through 2019 for physical activity and health is 0.46 articles per 100,000 inhabitants. The rate has increased as follows: 0.0005 in the 1960s, 0.0005 in the 1970s, 0.0045 in the 1980s, 0.0188 in the 1990s, 0.0872 in the 2000 decade and 0.2461 per 100,000 inhabitants in the 2010 decade. This represents an exponential growth with 700% increase by 1980, 3478% increase by 1990, 16,525% increase by 2000 and 46,843% increase by 2010. When comparing the rates between decades we observed a ten-fold increase from 1960 to 1980, four-fold increase from 1980 to 1990, five-fold increase from 1990 to 2000 and three-fold increase up to 2019 (data not shown in tables).

**Fig. 2:**
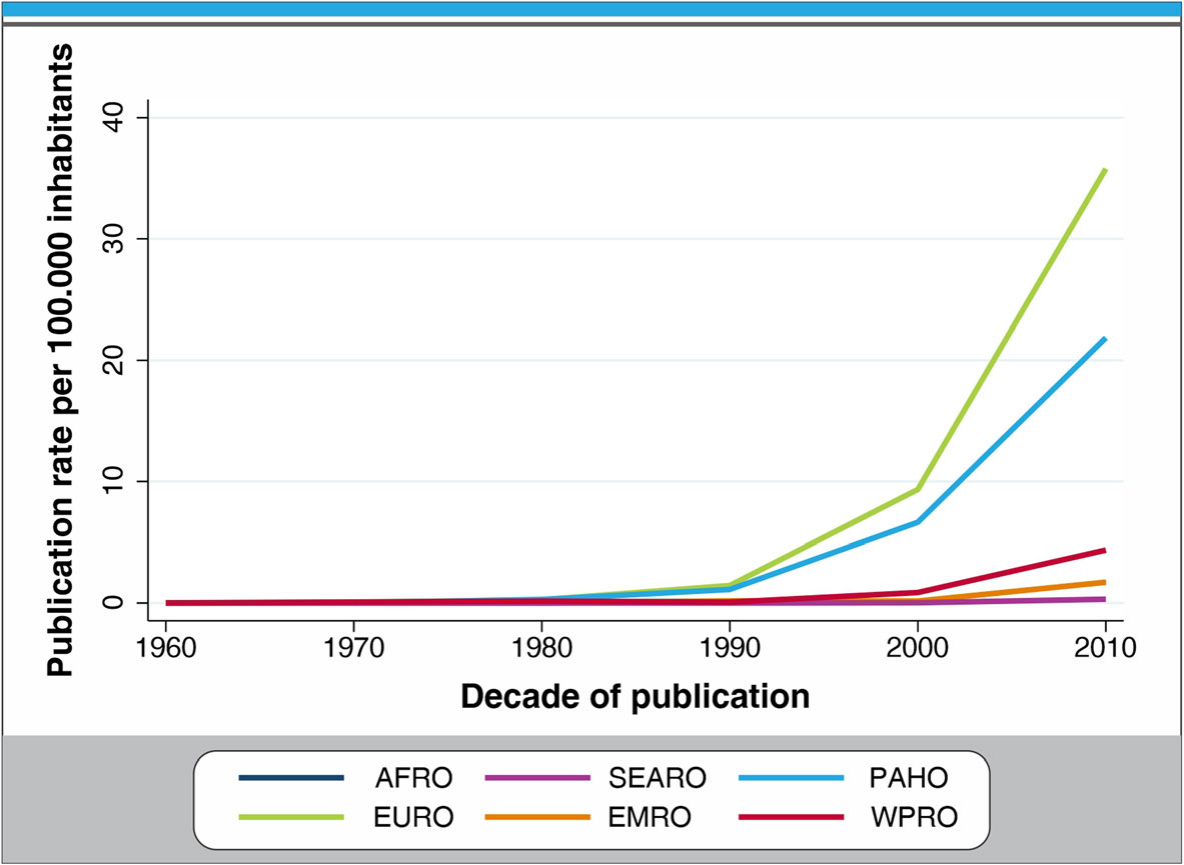
Publication rate per 100,000 inhabitants by region, 1950–2019

**Fig. 3:**
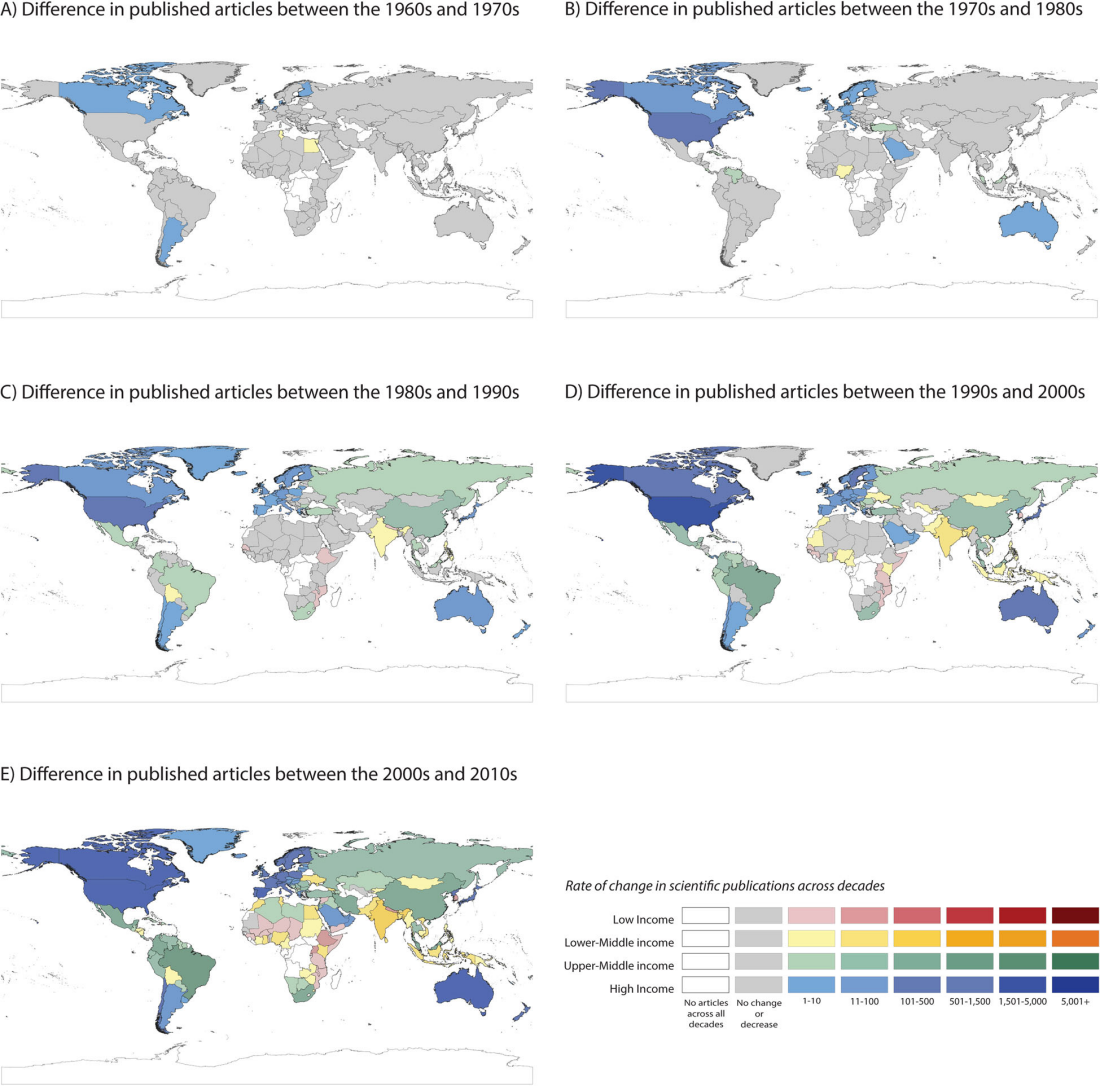
Worldwide time trends in physical activity research productivity, 1950–2019

Country-specific publication rates per 100,000 inhabitants by decade varied widely from less than 0.004 to 16.3 articles per 100,000 inhabitants (supplementary appendix,  webtable 1). When comparing regions, it was observed that publication rates increased slowly before the 1990’s and more rapidly in the period between 2000 and 2010, particularly in Europe. SEARO and AFRO have steady patterns with similar rates of increase over time. EURO was the region with the highest publication rate (1.44 articles per 100,000 inhabitants) and SEARO had the lowest rate (0.04 articles per 100,000 inhabitants). Regional time trends show an increasing pattern for all regions. EURO and PAHO rates are greater in all decades when compared to the rest of the regions. The WPRO 2000–2010 trend resembles the PAHO 1990–2000 trend. EMRO and AFRO 1990–2010 trends resemble EURO and PAHO 1970–1990 trends (Figs. 2 and 3).

PAHO was the most productive region in the world in absolute terms with more than ten thousand articles. Thirty-four out of the 44 countries in the region contributed at least one article. The United States (1st), Canada (2nd) and Brazil (4th) drive the region’s high publication rate and are all among the top five countries in total publications. The overall publication rate per 100,000 inhabitants was 1.43 articles per 100,000 inhabitants. It was one of the two regions with any publications before 1970 (Webtable 1 and Figs. 2 and 3). The United States is the leading country in scientific production for PA, contributing 6111 articles, 25.6%, of the total publications from 1950 to 2016. Brazil contributed 1200 articles, 5% of the total publications over the study period and was the only upper middle income country among the ten most productive nations.

EURO was the second most productive region in the world with more than eight thousand articles from 50 of the 62 countries in the region. The overall publication rate per 100,000 inhabitants was 1.44 articles per 100,000 inhabitants with a sharply increasing trend by decade. This region had several countries among the ten most productive nations for physical activity research: The Netherlands, Spain, Sweden, England, Germany and Finland.

WPRO was among the leaders in total publications with 3492 articles from 30 out of the 31 countries in the region. Australia was the third most productive country worldwide. The overall publication rate per 100,000 inhabitants was 0.23 articles per 100,000 inhabitants and the trend by decades of publication increased over time. China was one of the two upper middle income countries in the global top ten of publications.

In SEARO we observed the greatest inequalities between regional population and research output. Ten of the eleven countries in the region contributed published research (2.68% of the total global research publications from 1950 to 2019 from a region that comprises 26% of global population). Articles came predominantly from India (40%). The mean publication rate per 100,000 inhabitants was 0.04 articles per 100,000 inhabitants and the trend by decades for publication showed a slight increase in the 2000’s. Publication rates have been steady and low over time compared with regions with similar low productivity such as AFRO and EMRO.

The EMRO and AFRO regions had 0.13 and 0.09 articles per 100,000 inhabitants, respectively. Iran and South Africa were the most productive in their respective regions. Publication rates were constantly increasing over time for AFRO, when compared with EMRO that had fluctuating patterns. Countries with no published research in the field are listed in Webtable 1.

Publication rates by country income level showed that high income countries had the highest publication rates per 100,000 inhabitants, with an average of 9.28 articles per 100,000 inhabitants. In contrast, among upper middle, lower middle, and low income countries, the average number of publications per 100,000 inhabitants was less than 1. The Chi square test for trend was not significant *p* > 0.05., The Fisher-Hayter pairwise comparisons test for trend of publication rate by income level was significant p<0.005.

**Fig. 4:**
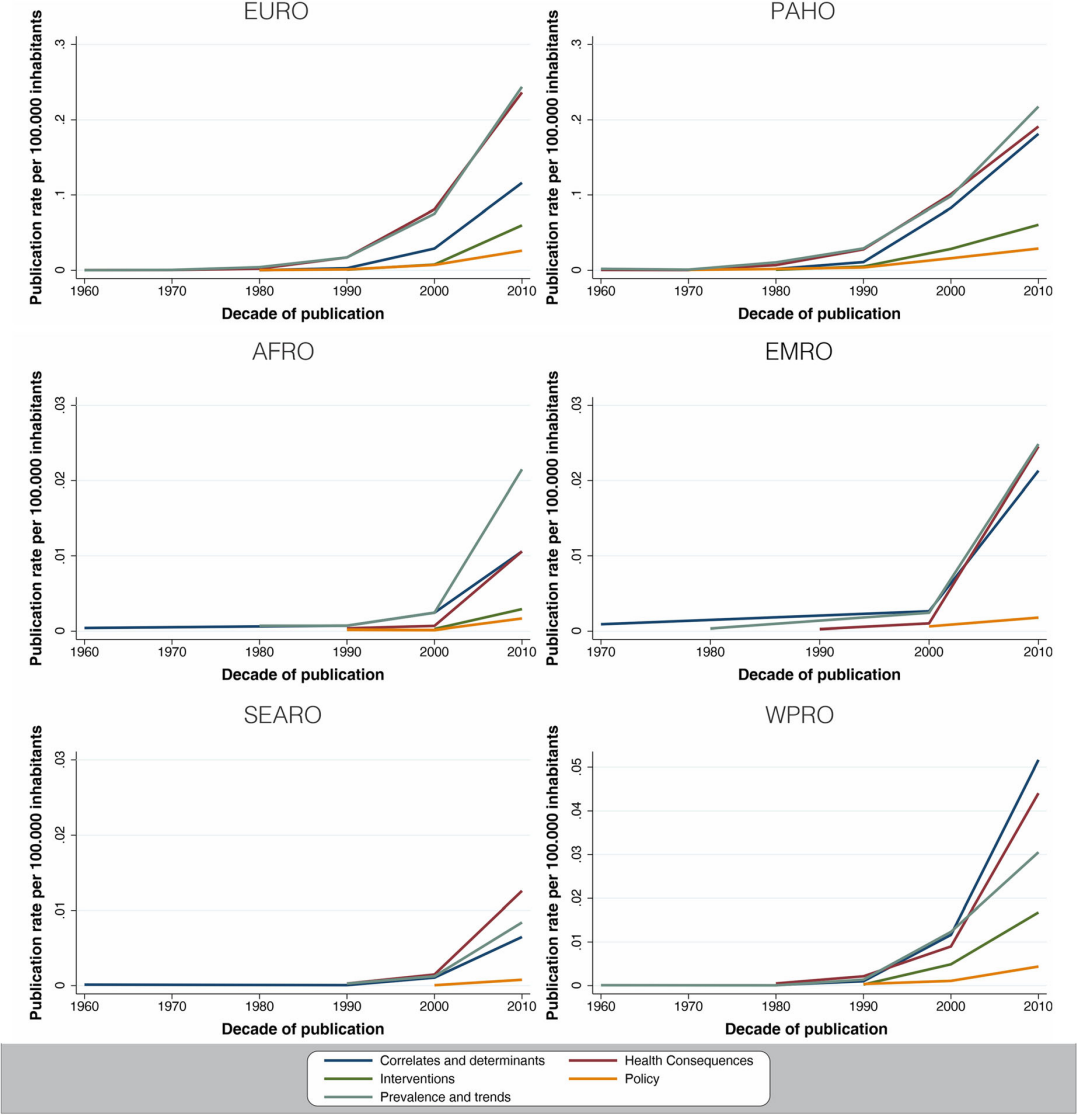
Publication rate per 100,000 inhabitants by decade of publication by study type by world regions

Figures 3 and 4 show the increase in the number of articles when comparing decades. High income countries achieved as a whole an increase of 5000+ articles between the 2000s and 2010s decades, compared to low income countries that achieved a maximum increase of 101–500 articles in the same period of time.

#### Types of physical activity publications

Overall, of the 23,860 articles, 82.5% were observational and 17.5% were intervention studies. Of the observational studies 83.9% were cross-sectional, 14.1% longitudinal and 2.0% case-control studies. Almost half (47.8%) publications included more than one age group, 26.32% publications focused on children and adolescents, 14.2% on adults and less than 10% on older adults.

The distribution by study type was: 32.5% prevalence measurements and trends, 31.7% health consequence, 23.2% correlates and determinants, 8.3% interventions and 3.9% policy studies. Approximately 17.3% of the studies reported using objective, device-based physical activity measures (e.g., accelerometers, pedometers). One out of ten studies 10.2% reported being part of a multicountry study (Table 3).

**Table 3:** Physical activity research characteristics by world region and income classification, 1950–2016

	***World region***^a^ ***n(%)***						***Income group***^a^ ***n(%)***			
	Africa (AFRO)	Eastern Mediterranean (EMRO)	Europe (EURO)	South East Asia (SEARO)	The Americas and the Caribbean (PAHO)	Western Pacific (WPRO)	High Income (HI)	Upper middle income (UMI)	Lower middle income (LMI)	Low income (LI)
**Total number of articles (n = 23,860)**	514	524	8370	639	10,321	3492	19,144	3534	915	267
Study design										
Observational	475 (92.4)	447 (85.3)	6866 (82.0)	535 (83.7)	8505 (82.4)	2851 (81.6)	15,649 (81.7)	2987 (84.5)	791 (86.5)	252 (94.4)
Experimental	39 (7.6)	77 (14.7)	1504 (18.0)	104 (16.3)	1816 (17.6)	641 (18.4)	3495 (18.3)	547 (15.5)	124 (13.6)	15 (5.6)
Study’s population age group										
Adult population > =18 years < 60 years	79 (15.4)	117 (22.3)	1203 (14.4)	96 (15.0)	1678 (16.3)	370 (10.6)	2717 (14.2)	637 (18.0)	157 (17.2)	32 (12.0)
Children and adolescents < 18 years	143 (27.8)	119 (22.7)	2509 (30.0)	125 (19.6)	2528 (24.5)	857 (24.5)	5146 (26.9)	867 (24.5)	209 (22.8)	59 (22.1)
Specific for older adults population > =60 years	15 (2.9)	13 (2.5)	809 (9.7)	44 (6.9)	885 (8.6)	418 (12.0)	1800 (9.4)	339 (9.6)	34 (3.7)	11 (4.1)
More than one age group	268 (52.1)	262 (50.0)	3660 (43.7)	368 (57.6)	5052 (49.0)	1792 (51.3)	9104 (47.6)	1638 (46.4)	499 (54.5)	161 (60.3)
Studies including pregnant women	10 (2.0)	12 (2.3)	152 (1.8)	10 (1.6)	206 (2.0)	61 (1.8)	352 (1.8)	74 (2.1)	21 (2.3)	4 (1.5)
Study type classification										
Prevalence, measurement & trends	232 (45.1)	167 (31.9)	3068 (36.7)	186 (29.1)	3316 (32.1)	802 (23.0)	6112 (31.9)	1174 (33.2)	357 (39.0)	128 (47.9)
Correlates & determinants	125 (24.3)	147 (28.1)	1337 (16.0)	144 (22.5)	2609 (25.3)	1174 (33.6)	4367 (22.8)	910 (25.8)	220 (24.0)	39 (14.6)
Health consequences	109 (21.2)	158 (30.2)	3033 (36.2)	270 (42.3)	3036 (29.4)	1011 (29.0)	6166 (32.2)	1098 (31.1)	266 (29.1)	87 (32.6)
Interventions	30 (5.8)	38 (7.3)	619 (7.4)	23 (3.6)	890 (8.6)	399 (11.4)	1724 (9.0)	220 (6.2)	46 (5.0)	9 (3.4)
Policy	18 (3.5)	14 (2.7)	313 (3.7)	16 (2.5)	470 (4.6)	106 (3.0)	775 (4.1)	132 (3.7)	26 (2.8)	4 (1.5)
Study topic										
Cardiovascular disease	82 (16.0)	81 (15.5)	1122 (13.4)	130 (20.3)	1221 (11.8)	301 (8.6)	2237 (11.7	481 (13.6)	164 (18.0)	55 (20.6)
Cancer	10 (2.0)	18 (3.4)	330 (3.9)	15 (2.4)	539 (5.2)	128 (3.7)	930 (4.9)	80 (2.3)	22 (2.4)	8 (3.0)
Mental health and illness	23 (4.5)	16 (3.1)	482 (5.8)	47 (7.4)	528 (5.1)	219 (6.3)	1079 (5.6)	170 (4.8)	42 (4.6)	24 (9.0)
Earth/environmental/atmospheric sciences	1 (0.2)	3 (0.6)	36 (0.4)	1 (0.2)	38 (0.4)	17 (0.5)	84 (0.4)	11 (0.3)	1 (0.1)	0 (0.0)
Built and natural environment	18 (3.5)	24 (4.6)	433 (5.2)	24 (3.8)	802 (7.8)	346 (9.9)	1370 (7.2)	239 (6.8)	34 (3.7)	4 (1.5)
Sedentary time (different than TV time only)	9 (1.8)	10 (1.9)	196 (2.3)	14 (2.2)	228 (2.2)	78 (2.2)	386 (2.0)	131 (3.7)	16 (1.8)	2 (0.8)
Population with physical disabilities	3 (0.6)	3 (0.6)	185 (2.2)	3 (0.5)	201 (2.0)	39 (1.1)	390 (2.0)	41 (1.2)	1 (0.1)	2 (0.8)
Nutrition	72 (14.0)	59 (11.3)	874 (10.4)	46 (7.2)	1029 (10.0)	257 (7.4)	1683 (8.8)	521 (14.7)	97 (10.6)	36 (13.5)
Methods	46 (9.0)	15 (2.9)	654 (7.8)	24 (3.8)	915 (8.9)	400 (11.5)	1734 (9.1)	259 (7.3)	54 (5.9)	7 (2.6)
International policy documents and recommendations	24 (4.7)	14 (2.7)	322 (3.9)	16 (2.5)	491 (4.8)	135 (3.9)	832 (4.4)	141 (4.0)	25 (2.7)	4 (1.5)
Healthy lifestyle studies	10 (2.0)	42 (8.0)	307 (3.7)	26 (4.1)	279 (2.7)	151 (4.3)	565 (3.0)	205 (5.8)	38 (4.2)	7 (2.6)
Other	216 (42.0)	239 (45.6)	3429 (41.0)	293 (45.9)	4050 (39.2)	1421 (40.7)	7854 (41.0)	1255 (35.5)	421 (46.0)	118 (44.2)
Studies using physical activity objective measures	72 (14.0)	23 (4.4)	1674 (20.0)	39 (6.1)	1752 (17.0)	567 (16.2)	3720 (19.4)	310 (8.8)	80 (8.7)	17 (6.4)
Study including multiple countries	111 (21.6)	29 (5.5)	1485 (17.7)	77 (12.1)	503 (4.9)	218 (6.2)	1856 (9.7)	386 (10.9)	156 (17.1)	25 (9.4)

^a^ Database search was conducted for the 217 world countries GoPA! list. Population, world region and income group classifications according to the World Bank. United Kingdom was divided in its 4 countries (England, Scotland, Wales and Northern Ireland)

The most common topics studied included physical activity and: 12.3% cardiovascular disease, 9.8% nutrition, 8.6% methods, 6.9% built and natural environment, 5.5% mental health and illness, 4.4% cancer, 4.2% international policy documents and recommendations and, 3.4% healthy lifestyles. The least studied topics were 2.2% sedentary time, 1.8% disabilities and 0.4% earth/environmental/atmospheric sciences (Data not shown in tables).

As presented in Fig. 4, EURO and PAHO are the regions that first conducted research in physical activity. Prevalence, correlates and determinants and health consequences studies are the most frequently conducted in all regions, supplementary appendix figures 1, 2. However, AFRO, SEARO and EMRO show an increased rate from the 1990s onward. The publication rates for the policy study type are the lowest and most homogeneous across all study type trends, meaning that policy was the least published area across all regions, supplementary appendix figures 4–8 show time trends according to study type.

## Discussion

To our knowledge, this is the first systematic review of the trends and patterns of published research on physical activity since the inception of the field in 1950. Our analysis suggests several key findings: 1) The field of physical activity research has grown tremendously worldwide in the last 60 years with a 46,843% increase in publication rate and more than 80% of the world’s countries with at least one publication on physical activity since 1950; 2) The field of physical activity research has evolved over time to become much more diverse in study types, disciplines engaged in research, geographic areas where studies are conducted, research methodologies, and population groups included in the studies; 3) Large inequities exist across geographic regions, income groups and countries in physical activity research productivity; 4) The identified trends and patterns provide information for identifying research gaps and guiding actions to optimize conduct and translation of research into physical activity policy, promotion, and surveillance at the national, regional, and global levels.

The physical activity research growth pattern described in the literature is consistent with growing scientific productivity for most countries and disciplines over time [27–29]. Although the number of physical activity publications is a fraction of scientific publications in many other areas, supplementary appendix figure 9 shows that the pattern of exponential increase in physical activity research is similar to that for other healthy lifestyle research areas and also followed traditional disease oriented research (NCD, Cancer, CVD) patterns up until 1990 at which point physical activity and other “lifestyle” research began to grow at a much faster rate. A study conducted from 1981 to 1994 with publications from the Institute of Scientific Information, shows similar patterns in terms of the most productive countries in research. For example, the United States had a dominant position in research, accounting for 35% of published research followed by the United Kingdom, Japan, Canada, Germany, France, Italy, India, Australia and the Netherlands [27–29].

Physical activity research has developed over time including more diversity in study types, disciplines engaged in research, geographic areas where studies are conducted, research methodologies and population groups included in the studies. This could reflect a more mature research field evolving from a strong health science focus based in North American and Europe towards global and diverse studies. These findings are consistent with a recent network analysis of physical activity and health publications [26].

Despite this, in all regions and worldwide, 82.5% of the studies are observational (mostly cross-sectional), one third are about health consequences, and fewer than 5% are about physical activity policy, which is alarming. Such pattern of research outputs may reflect: 1) lack of appropriate funding and incentives to conduct more studies with more complex designs like longitudinal, intervention and policy studies and, 2) local capacity, expertise and training limitations that constrain the ability to gain funding to implement these types of studies [30, 31]. This persistent focus on research areas that already have enough evidence (e.g physical activity for physical health; and individual- and inter-personal level correlates and determinants and built environment correlates more recently) may be compromising much needed attention to other critical study areas where evidence is more scarce (e.g., intervention, policy) [31]. A possible explanation for this is that in countries where the field started, there was and continues to be more focus on linking physical activity to health outcomes as described by the systematic framework to classify phases of research on health promotion and disease prevention [32]. However, it is not necessary for countries that started conducting research decades later to conduct the same studies, not only because innovative and context-tailored studies are needed, but also because there is a need to conduct policy and intervention studies worldwide. In Latin America for example, most articles were published in recent decades with little research (beyond Brazil) on the health effects of physical activity and most studies related to built environment correlates and regional surveillance (many as part of multi-country studies). This is an example of scientific production building on cumulative knowledge produced in other regions without replicating the same evolutionary pattern of studies.

**Fig. 5:**
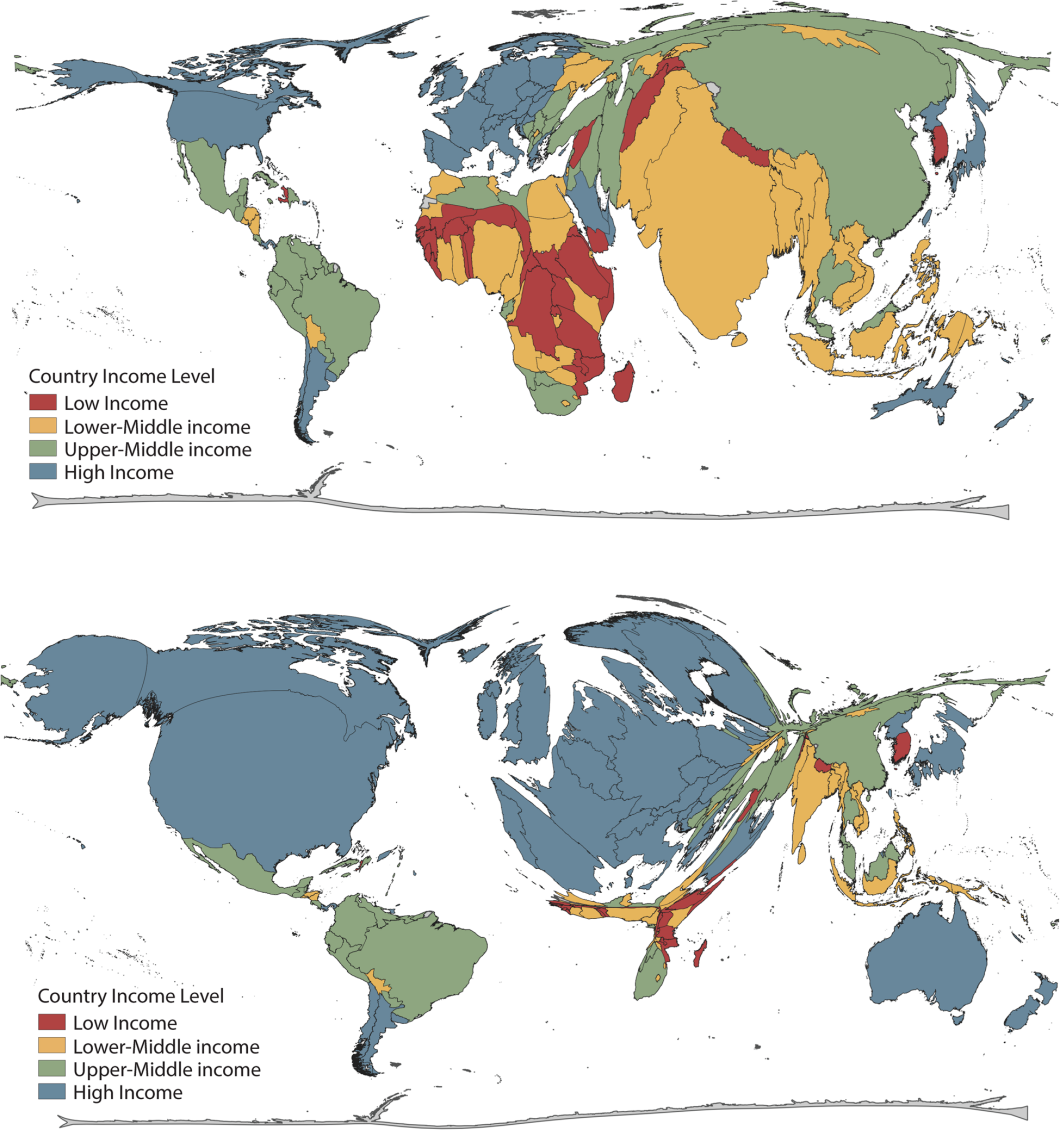
Mismatch between world population and physical activity scientific publications from 1950 to 2019 about here. Legend: Countries in this density equalizing map are resized according to the average population between 1950 and 2019 and the total number of physical activity articles between 1950 and 2019 according to the Gastner and Newman algorithm

An uneven distribution of research productivity by region and country income group was found, with countries with the highest burden of deaths due to non-communicable diseases attributable to physical inactivity having low research productivity ( Supplementary appendix, webtable 1) [33]. There is a 50-fold difference in publications per 100,000 inhabitants between high and low-income countries, suggesting a substantial gap in the knowledge base between the most scientifically active countries and others [34, 35]. Ten percent of the world’s population lives in the five countries with the highest productivity contributing more than 47% of the research (United States, Canada, Australia, Brazil, Netherlands). While, 81.5% of countries had at least one physical activity and health publication, two WHO regions (PAHO (48.4%) and EURO (34.8%)) accounted for more than 78% of publications. In 2015, GoPA! showed similar patterns with HICs having the largest share of worldwide publications as of 2013 [12]. Even though UMICs and LICs increased their productivity, the gap between LICs and HICs was more than 50-fold. Publications are highly concentrated in few countries, leaving most of the world’s population without local scientific evidence. This scarcity of local evidence geographically overlaps with countries with the highest burden of non-communicable diseases, no periodic or active physical activity surveillance and no stand-alone physical activity policies [12, 30, 31]. Figure 5 shows the mismatch between world population and physical activity publications [36].

Consistent with our findings, literature shows that scientific publications on health topics were disproportionately distributed and highly concentrated among the world’s richest countries [34, 37–42], altogether contributing to at least 70% of the scientific production [38]. This may be explained by these highly productive countries having both the largest economies in the world and more investment in research and development (R&D). A high investment in R&D can lead to more highly trained researchers, better research incentives and may translate into better knowledge production, increased availability of resources and capacity to build local research teams [43]. Webtable 1 shows the R&D investment by country.

Despite the high concentration of articles in few countries, we also found that more countries worldwide showed an increase in the number of publications in the last 2 decades. Figure 3 and appendix figure 3–8 show changes in research productivity by country and income group. For example, when comparing productivity in the 1950–1990 and 2000–2019 periods, Colombia, Poland and Brazil increased by 100 times their scientific output, followed by Saudi Arabia, Thailand, South Africa that increased it by at least 70 fold, and Portugal, Ireland and New Zealand that increased it by 50 fold. These findings may reflect an increase in local capacity and/or cross-country collaboration, both identified as important factors for enhancing research quality and productivity. Participation in large-scale international studies and consortia can boost scientific productivity and help build local capacity in the least developed countries [44, 45, 46].

The identified trends and patterns provide information for closing research gaps and guiding actions to optimize the translation of research into physical activity policy, promotion, and surveillance at the national, regional, and global levels. Physical activity surveillance, policy and research have been observed to be positively correlated, thus enhancing research may advance much needed public health policy and promotion in low and middle income countries [6]. The speed at which research has to grow in these settings to begin to close the gap with high-income countries is daunting. However, increasing collaboration with HIC researchers and global networks may be a feasible strategy for reaching better global research equity for PA. A greater understanding is needed of the structural determinants of the research inequalities found in this study and pathways for building local research capacity and knowledge translation. Future studies and research designs may include measures such as quality and impact evaluation of research, scalability, and knowledge translation that can contribute to a more complete understanding of research development in this field beyond simple research quantity. 

### Limitations

Results should be interpreted with caution given the following limitations: 1) This review focused on publications that included a title or abstract in English, Spanish and Portuguese (the working group was fluent in Spanish and Portuguese as well as English). Given that most of the articles were in English, the potential for an English language bias exists. It has been observed that publishing scientific articles in a language other than English may be a barrier to retrieving them in systematic searches. This has been documented for publications in Russian, Japanese and Chinese [28, 29]. We may have under-reported publication productivity for countries in which publication is common in a language other than English, and may have over- reported the productivity of the Latin American region, Portugal and Spain, compared with the rest of the non-English speaking world. However, given the limited resources and the scope of the project (ten researchers), it was not possible for us to include all publications of any language; 2) The search only included indexed publications and it is possible that grey literature was missed, therefore we may have systematically underestimated the volume of policy research, which may be more likely than other research, to be published as grey literature; 3) Peer-reviewed publications are generally regarded as the most important metric for measuring research productivity, however, there are other metrics (e.g. Bibliometrics and Altmetrics, research grants, and publication of books and evidence-based guidelines among others) that we did not assess that might provide additional depth to the analyses.

## Conclusion

This study estimated for the first time a total worldwide publication rate of 0.46 articles per 100,000 inhabitants with exponential growth from 1950 to the present. Despite most countries having at least one physical activity publication, the knowledge gap between geographic regions and by country income groups remains substantial. The least productive and poorest regions have productivity rates resembling those from previous decades in the richest and most productive countries, reflecting the heterogeneous evolution of the field of physical activity research. Focusing on the public health impact and global equity of research conducted in the coming years will be important for making all of the world more active.

## Supplementary Information

**Additional file 1: Supplementary appendix**. **Webtable 1**. Physical activity research characteristics per country, 1950–2019. **Appendix Figure 1**. Publication rate per 100.000 inhabitants by decade of publication by study design. **Appendix Figure 2**. Publication rate per 100,000 inhabitants by decade of publication by study’s population age group by world regions. **Appendix Figure 3**. Worldwide time trends in physical activity research by income group, 1950–2019. **Appendix Figure 4**. Worldwide time trends in physical activity prevalence measurement and trends research, 1950–2019. **Appendix Figure 5**. Worldwide time trends in physical activity correlates and determinants research, 1950–2019. **Appendix Figure 6**. Worldwide time trends in physical activity health consequences research, 1950–2019. **Appendix Figure 7**. Worldwide time trends in physical activity intervention research, 1950–2019. **Appendix Figure 8**. Worldwide time trends in physical activity policy research, 1950–2019. **Appendix Figure 9**. Worldwide research productivity of fields related to physical activity since 1950.

## Data Availability

The datasets used and/or analyzed during the current study are available from the corresponding author on reasonable request.

## Abbreviations

AFRO: Africa
EMRO: Eastern Mediterranean
EURO: Europe
PAHO: The Americas and the Caribbean
SEARO: South-East Asia
WPRO: Western Pacific
HICs: High income countries
UMICs: Upper middle income countries
LMICs: Lower middle income countries
LICs: Low income countries
PA: Physical activity

## References

[1] WHO. World Health Report: Reducing risks and promoting a healthy life. World Health Organization. 2002. Available from: http://www.who.int/whr/2002/en/. Accessed March 202.

[2] WHO. Global Status Report on noncommunicable diseases 2010. World Health Organization 2010. Available from: http://www.who.int/nmh/publications/ncd_report_full_en.pdf. Accessed March 2020.

[3] WHO. World Health Organization Global action plan for the prevention and control of noncommunicable diseases 2013–2020. 2013. Available from: https://www.who.int/nmh/events/ncd_action_plan/en/ Accessed March 2020.

[4] WHO. Global status report on noncommunicable diseases 2014; Available from: http://www.who.int/nmh/publications/ncd-status-report-2014/en/. Accessed March 2020.

[5] Guthold R, Stevens GA, Riley LM, Bull FC. Worldwide trends in insufficient physical activity from 2001 to 2016: a pooled analysis of 358 population-based surveys with 1· 9 million participants. Lancet Global Health. 2018;6(10). 10.1016/S2214-109X(18)30357-7. 10.1016/S2214-109X(18)30357-7 30193830

[6] Varela AR, Salvo D, Pratt M, Milton K, Siefken K, Bauman A (2018). Worldwide use of the first set of physical activity Country Cards: The Global Observatory for Physical Activity-GoPA!. Int J Behav Nutr Phys Act.

[7] WHO. ACTIVE: a technical package for increasing physical activity. Geneva: World Health Organization. 2018. Available from: https://apps.who.int/iris/handle/10665/275415; Accessed March 2020.

[8] Brownson RC, Baker EA, Leet TL, Gillespie KN, True WR (2010). Evidence-based public health: Oxford University press.

[9] Brownson RC, Royer C, Ewing R, McBride TD (2006). Researchers and policymakers: travelers in parallel universes. Am J Prev Med.

[10] GoPA! Global Observatory for Physical Activity 2016; Available from: http://www.globalphysicalactivityobservatory.com/goals/. Accessed March 2020.

[11] Pratt M, Sarmiento OL, Montes F, Ogilvie D, Marcus BH, Perez LG (2012). The implications of megatrends in information and communication technology and transportation for changes in global physical activity. Lancet.

[12] Ramirez Varela A, Pratt M, Powell K, Lee IM, Bauman A, Heath G, et al. Worldwide surveillance, policy and research on physical activity and health: the global Observatory for Physical Activity - GoPA! J Phys Act Health. 2017:1–28. 10.1123/jpah.2016-0626. 10.1123/jpah.2016-0626 28513338

[13] Moher D, Liberati A, Tetzlaff J, Altman DG (2009). Preferred reporting items for systematic reviews and meta-analyses: the PRISMA statement. Ann Intern Med.

[14] Chien PFW, Khan KS, Siassakos D (2012). Registration of systematic reviews: PROSPERO. BJOG Int J Obstet Gynaecol.

[15] Trowbridge MJ, Schmid TL (2013). Built environment and physical activity promotion: place-based obesity prevention strategies. J Law Med Ethics.

[16] World Bank. List of countries and economies 2014; Available from: http://data.worldbank.org/country. Accessed March 2020.

[17] WHO. Global recommendations on physical activity for health. World Health Organization. 2015; Available from: http://www.who.int/dietphysicalactivity/factsheet_recommendations/en/. Accessed March 2020. 26180873

[18] WHO. Global health observatory data repository, World Health Organization; 2014 Available from: http://www.who.int/gho/en/. Accessed March 2020.

[19] Northern Ireland Statistics and Research Agency NISRA. Mid Year Population Estimates 2018; Available from: https://www.nisra.gov.uk/statistics/population/mid-year-population-estimates. Accessed March 2020.

[20] National records of Scotland. Scotland Mid-Year Population Estimates 2018; Available from: https://www.nrscotland.gov.uk/statistics-and-data/statistics/statistics-by-theme/population/population-estimates/mid-year-population-estimates. Accessed March 2020.

[21] Statistics for Wales. Wales population mid-year estimate 2018; Available from: https://statswales.gov.wales/Catalogue/Population-and-Migration/Population/Estimates/Local-Authority/populationestimates-by-localauthority-year. Accessed March 2020.

[22] Office for National Health Statistics. England population mid-year estimate 2018; Available from: https://www.ons.gov.uk/peoplepopulationandcommunity/populationandmigration/populationestimates/datasets/populationestimatesforukenglandandwalesscotlandandnorthernireland. Accessed March 2020.

[23] WHO. Health in 2015: from MDGs, Millennium Development Goals to SDGs, Sustainable Development Goals. World Health Organization; 2015. Available from: http://apps.who.int/iris/bitstream/10665/200009/1/9789241565110_eng.pdf?ua=1. Accessed March 2020.

[24] ISPAH International Society for Physical Activity and Health (2017). The Bangkok declaration on physical activity for Global Health and sustainable development. Br J Sports Med.

[25] Bull FC, Gauvin L, Bauman A, Shilton T, Kohl HW, Salmon A (2010). The Toronto charter for physical activity: a global call for action. J Phys Act Health.

[26] Varela AR, Pratt M, Harris J, Lecy J, Salvo D, Brownson RC (2018). Mapping the historical development of physical activity and health research: a structured literature review and citation network analysis. Prev Med.

[27] Jaffe K, ter Horst E, Gunn LH, Zambrano JD, Molina G. A network analysis of research productivity by country, discipline, and wealth. Plos one. 2020;15(5). 10.1371/journal.pone.0232458. 10.1371/journal.pone.0232458 PMC7219709 32401823

[28] King DA (2004). The scientific impact of nations. Nature..

[29] May RM (1997). The scientific wealth of nations. Science..

[30] Pratt M, Varela AR, Salvo D, Kohl Iii HW, Ding D. Attacking the pandemic of physical inactivity: what is holding us back? Br Assoc Sport Exerc Med. 2020. 10.1136/bjsports-2019-101392. 10.1136/bjsports-2019-101392 31704698

[31] Ding D, Varela AR, Bauman AE, Ekelund U, Lee I-M, Heath G (2020). Towards better evidence-informed global action: lessons learnt from the lancet series and recent developments in physical activity and public health. Br J Sports Med.

[32] Sallis JF, Owen N, Fotheringham MJ (2000). Behavioral epidemiology: a systematic framework to classify phases of research on health promotion and disease prevention. Ann Behav Med.

[33] Lee IM, Shiroma EJ, Lobelo F (2012). Effect of physical inactivity on major non-communicable diseases worldwide: an analysis of burden of disease and life expectancy. Lancet.

[34] Perez-Iratxeta C, Andrade MA. Worldwide scientific publishing activity. Science. 2002;297(5581):519-. doi: 10.1126/science.297.5581.519b. 10.1126/science.297.5581.519b 12143877

[35] Van Noorden R. Global scientific output doubles every nine years. Nat News Blog. 2014.

[36] Gastner MT, Newman ME (2004). Diffusion-based method for producing density-equalizing maps. Proc Natl Acad Sci.

[37] Bliziotis IA, Paraschakis K, Vergidis PI, Karavasiou AI, Falagas ME (2005). Worldwide trends in quantity and quality of published articles in the field of infectious diseases. BMC Infect Dis.

[38] Cash-Gibson L, Rojas-Gualdrón DF, Pericàs JM, Benach J. Inequalities in global health inequalities research: A 50-year bibliometric analysis (1966–2015). PloS one. 2018;13(1). 10.1371/journal.pone.0191901. 10.1371/journal.pone.0191901 PMC5792017 29385197

[39] Grant C, Williams B, Driscoll T (2018). Historical trends in publications in the international journal of epidemiology. Int J Epidemiol.

[40] Herlihy N, Bar-Hen A, Verner G, Fischer H, Sauerborn R, Depoux A, et al. Climate change and human health: what are the research trends? A scoping review protocol. BMJ Open. 2016;6(12). 10.1136/bmjopen-2016-012022. 10.1136/bmjopen-2016-012022 PMC5223655 28011805

[41] Hofman KJ, Kanyengo CW, Rapp BA, Kotzin S (2009). Mapping the health research landscape in sub-Saharan Africa: a study of trends in biomedical publications. J Med Libr Assoc.

[42] Paraje G, Sadana R, Karam G (2005). Increasing international gaps in health-related publications. Science.

[43] OECD. Gross domestic spending on R&D (indicator). Organisation for economic co-operation and development; 2020. Available from: https://data.oecd.org/rd/gross-domestic-spending-on-r-d.htm. Accessed March 2020.

[44] Kwiek M (2015). The internationalization of research in Europe: A quantitative study of 11 national systems from a micro-level perspective. J Stud Int Educ.

[45] Hallal PC, Andersen LB, Bull FC, Guthold R, Haskell W, Ekelund U (2012). Global physical activity levels: surveillance progress, pitfalls, and prospects. Lancet..

[46] Sallis JF, Bull F, Guthold R, Heath GW, Inoue S, Kelly P, et al. Progress in physical activity over the Olympic quadrennium. Lancet. 2016. 10.1016/S0140-6736(16)30581-5. 10.1016/S0140-6736(16)30581-5 27475270

