# Correction to: Global, regional, and national trends and patterns in physical activity research since 1950: a systematic review

**DOI:** 10.1186/s12966-021-01100-3

**Published:** 2021-03-08

**Authors:** Andrea Ramírez Varela, Gloria Isabel Nino Cruz, Pedro Hallal, Cauane Blumenberg, Shana Ginar da Silva, Deborah Salvo, Rafaela Martins, Bruna Gonçalves Cordeiro da Silva, Eugen Resendiz, Maria Catalina del Portillo, Luciana Zaranza Monteiro, Selina Khoo, Kar Hau Chong, Marcelo Cozzensa da Silva, Alice Mannocci, Ding Ding, Michael Pratt

**Affiliations:** 1 School of Medicine, Universidad de los Andes, Cra 7 #116-05, 11001000 Bogotá, Colombia; 2 School of Physiotherapy, Universidad Industrial de Santander, Bucaramanga, Colombia; 3 Post-Graduate Program in Epidemiology, Federal University of Pelotas, Pelotas, Brazil; 4 Post-Graduate in Biomedical Science, School of Medicine, Federal University of Fronteira Sul, Chapecó, Brazil; 5 Prevention Research Center, Brown School, Washington University in St. Louis, St. Louis, USA; 6 University Center of the Federal District, Brasília, Brazil; 7 University of Malaya, Kuala Lumpur, Malaysia; 8 Early Start, University of Wollongong, Wollongong, Australia; 9 Mercatorum University, Rome, Italy; 10 University of Sydney, Sydney, Australia; 11 University of California San Diego Herbert Wertheim School of Public Health and Longevity Science, San Diego, USA

**Correction to: Int J Behav Nutr Phys Act (2021) 18:5**

**https://doi.org/10.1186/s12966-020-01071-x**

Following publication of the original article [1], the authors identified an error in the affiliation of Dr. Kar Hau Chong.

The incorrect affiliation: University of Malaya, Kuala Lumpur, Malaysia.

The correct affiliation: Early Start, University of Wollongong, Wollongong, Australia.

The author group has been updated above and the original article [1] has been corrected.
