# Supplementary material for: Global, regional, and national trends and patterns in physical activity research since 1950: a systematic review
Source: Int J Behav Nutr Phys Act. 2021 Jan 7;18:5. doi: 10.1186/s12966-020-01071-x (PMC7792158; doi:10.1186/s12966-020-01071-x)

**Supplementary appendix**

This is an online appendix with supplemental material for the article **: “Global, regional, and national trends and patterns in physical activity research since 1950: a systematic review” by** Andrea Ramirez Varela^1^, Gloria Isabel Nino Cruz^2^, Pedro Hallal^3^, Cauane Blumenberg^3^, Shana Ginar da Silva^3^, Deborah Salvo^4^, Rafaela Martins^3^, Bruna Gonçalves Cordeiro da Silva^3^, Eugen Resendiz^4^, Catalina del Portillo^1^, Luciana Zaranza Monteiro^5^, Selina Khoo^6^, Chong Kar Hau^6^, Marcelo Cozzensa^3^, Alice Manocci^7^, Ding Ding^8^, Michael Pratt^9^.

Table of Content

- Systematic review search terms. …………………………………………… 2
- Webtable 1 .......................................................................................................... 4
- Appendix Figure 1. Publication rate per 100.000 inhabitants by decade of publication by study design ……………………………………………………………….. 16
- Appendix Figure 2. Publication rate per 100,000 inhabitants by decade of publication by study`s population age group by world regions …………………………… 17
- Appendix Figure 3. Worldwide time trends in physical activity research by income group, 1950-2019 ………………………………………………………………. 18
- Appendix figure 4. Worldwide time trends in physical activity prevalence measurement and trends research, 1950-2019 ……………………………….. 19
- Appendix figure 5. Worldwide time trends in physical activity correlates and determinants research, 1950-2019 …………………………………………….. 20
- Appendix figure 6. Worldwide time trends in physical activity health consequences research, 1950-2019 …………………………………………………………… 21
- Appendix figure 7. Worldwide time trends in physical activity intervention research, 1950-2019 ……………………………………………………………………… 22
- Appendix figure 8. Worldwide time trends in physical activity policy research, 1950-2019 ……………………………………………………………………………. 23
- Appendix figure 9. Worldwide research productivity of fields related to physical activity since 1950 ……………………………………………………………. 24

# Systematic review search terms

## Search terms

The search terms for physical activity (in title or abstract) and country name in English (anywhere in the title, abstract, text or affiliation) were used. ‘Physical activity’ terms included both those referring to physical movement, as well as those encompassing the concept of sedentary behaviors different than TV viewing. The ‘physical activity’ search terms used were as follows: physical activity OR physically active OR physical inactivity OR physically inactive OR fitness OR exercis* OR walk OR walking OR sedentary OR active transport* OR active transit OR active travel OR commut* OR active commuting OR bicycle OR bicycling OR bike OR biking OR active living OR active-living**.**

PUBMED SEARCH

(after the search, select the studies on humans): 1950-2019

((physical activity OR physically active OR physical inactivity OR physically inactive OR fitness OR exercise* OR walk OR walking OR sedentary OR active transport* OR active transit OR active travel OR commut* OR active commuting OR bicycle OR bicycling OR bike OR biking OR active living OR active-living [Title/Abstract]) AND Malaysia) AND (&quot;1950/01/01&quot;[Date - Publication]: &quot;2016/12/31&quot;[Date-Publication])

SCOPUS SEARCH

TITLE-ABS-KEY ( &quot;physical AND activity&quot; )  OR  TITLE-ABS-KEY ( &quot;physical AND inactivity&quot; )  OR  TITLE-ABS KEY ( fitness )  OR  TITLE-ABS-KEY ( exercis )  OR  TITLE-ABS KEY ( walk )  OR  TITLE-ABS-KEY ( walking )  OR  TITLE-ABS-KEY ( sedentary )  OR  TITLE- ABS-KEY ( &quot;active AND transport&quot; )  OR  TITLE-ABS-KEY ( &quot;active AND living&quot; )  OR  TITLE-

ABS-KEY ( &quot;active AND transit&quot; )  OR  TITLE-ABS-KEY ( &quot;active AND travel&quot; )  OR  TITLE- ABS-KEY ( commut )  OR  TITLE-ABS KEY ( &quot;active AND commuting&quot; )  OR  TITLE-ABS- KEY ( bicycling )  OR  ( biking )  AND  TITLE-ABS- KEY ( malaysia )  AND  PUBYEAR  &gt;  1950  AND  ( LIMIT TO ( SUBJAREA ,  &quot;MEDI&quot; )  OR  LIMIT TO ( SUBJAREA ,  &quot;SOCI&quot; )  OR  LIMIT-

TO ( SUBJAREA ,  &quot;ENVI&quot; )  OR  LIMIT-TO ( SUBJAREA ,  &quot;NURS&quot; )  OR  LIMIT-

TO ( SUBJAREA ,  &quot;HEAL&quot; )  OR  LIMIT-TO ( SUBJAREA ,  &quot;EART&quot; )  OR  LIMIT-

TO ( SUBJAREA ,  &quot;PSYC&quot; )  OR  LIMIT-TO ( SUBJAREA ,  &quot;NEUR&quot; ) )  AND  ( LIMIT-

TO ( DOCTYPE ,  &quot;ar&quot; )  OR  LIMIT-TO ( DOCTYPE ,  &quot;re&quot; ) ) AND (EXCLUDE

(PUBYEAR,2017)

WEB OF SCIENCE SEARCH: **1950-2019**

((TS=(physical activity OR physically active OR physical inactivity OR physically inactive OR fitness OR exercis* OR walk OR walking OR sedentary OR active transport* OR active transit OR active travel OR commut* OR active commuting OR bicycle OR bicycling OR bike OR biking OR active living OR active-living))) AND Tipos de documento: (Article) Índices=SCI-EXPANDED, SSCI, A&amp;HCI, CPCI-S, CPCI-SSH, ESCI Tempo estipulado=1950-2016 AND ((TS=(Malaysia))) AND Tipos de documento: (Article) Índices=SCI-EXPANDED, SSCI, A&amp;HCI, CPCI-S, CPCI-SSH, ESCI Tempo estipulado=1950-2016 Update 2017 ate 2019 ((TS=(physical activity OR physically active OR physical inactivity OR physically inactive OR fitness OR exercis* OR walk OR walking OR sedentary OR active transport* OR active transit OR activetravel OR commut* OR active commuting OR bicycle OR bicycling OR bike OR biking OR active-living OR active living)))

| **Webtable 1. Physical activity research characteristics per country, 1950-2019** | | | | | | | | |
| --- | --- | --- | --- | --- | --- | --- | --- | --- |
| Income Group classification 2019**** | Region classification 2019*** | Country | GDP % spent in research and development 2020 | % Deaths by noncommunicable diseases attributable to physical inactivity | Population average | Number of Articles | Publication rate *100.000 | Country Contribution % |
| HIC | PAHO | United States | 2.84 | 10.8 | 250000000 | 6111 | 2.44 | 25.61 |
| HIC | PAHO | Canada | 1.57 | 9.1 | 28000000 | 1980 | 7.07 | 8.30 |
| HIC | WPRO | Australia | 1.87 | 10.1 | 18000000 | 1233 | 6.85 | 5.17 |
| UMIC | PAHO | Brazil | 1.26 | 13.2 | 180000000 | 1200 | 0.67 | 5.03 |
| HIC | EURO | Netherlands | 2.16 | 4.9 | 15000000 | 818 | 5.45 | 3.43 |
| HIC | EURO | Spain | 1.24 | 13.4 | 43000000 | 709 | 1.65 | 2.97 |
| HIC | EURO | England | 1.72 | - | 49000000 | 668 | 1.36 | 2.80 |
| HIC | EURO | Germany | 3.09 | 7.5 | 81000000 | 653 | 0.81 | 2.74 |
| HIC | EURO | Sweden | 3.34 | 11.8 | 9000000 | 645 | 7.17 | 2.70 |
| UMIC | WPRO | China | 2.19 | 8.3 | 1300000000 | 610 | 0.05 | 2.56 |
| HIC | WPRO | Japan | 3.26 | 16.1 | 130000000 | 575 | 0.44 | 2.41 |
| HIC | EURO | Finland | 2.77 | 10.1 | 5100000 | 523 | 10.25 | 2.19 |
| HIC | EURO | Denmark | 3.06 | 9.4 | 5300000 | 473 | 8.92 | 1.98 |
| HIC | EURO | Belgium | 2.82 | 11.4 | 10000000 | 435 | 4.35 | 1.82 |
| HIC | EURO | Norway | 2.07 | 11.8 | 4600000 | 397 | 8.63 | 1.66 |
| HIC | EURO | Italy | 1.40 | 14.6 | 56000000 | 357 | 0.64 | 1.50 |
| HIC | EURO | France | 2.20 | 8.7 | 63000000 | 317 | 0.50 | 1.33 |
| UMIC | WPRO | Malaysia | 1.44 | 16.4 | 23000000 | 286 | 1.24 | 1.20 |
| LMIC | SEARO | India | 0.65 | 4.2 | 970000000 | 259 | 0.03 | 1.09 |
| HIC | EURO | Portugal | 1.37 | 13.6 | 10000000 | 240 | 2.40 | 1.01 |
| HIC | EURO | Switzerland | 3.37 | - | 7300000 | 234 | 3.21 | 0.98 |
| HIC | WPRO | New Zealand | 1.37 | 12.7 | 4100000 | 232 | 5.66 | 0.97 |
| UMIC | PAHO | Mexico | 0.31 | 10.1 | 110000000 | 227 | 0.21 | 0.95 |
| HIC | EURO | Poland | 1.21 | 7.4 | 38000000 | 227 | 0.60 | 0.95 |
| UMIC | PAHO | Colombia | 0.24 | 11.7 | 43000000 | 226 | 0.53 | 0.95 |
| HIC | WPRO | Hong Kong SAR, China | 0.86 | - | 6400000 | 202 | 3.16 | 0.85 |
| HIC | EURO | Scotland | 0.36 | - | 5200000 | 192 | 3.69 | 0.80 |
| HIC | PAHO | Chile | 0.36 | - | 16000000 | 173 | 1.08 | 0.73 |
| HIC | EURO | Greece | 1.18 | 4.2 | 11000000 | 166 | 1.51 | 0.70 |
| UMIC | EMRO | Iran, Islamic Rep. | 0.83 | 9.9 | 78000000 | 157 | 0.20 | 0.66 |
| UMIC | AFRO | South Africa | 0.83 | 14 | 48000000 | 156 | 0.33 | 0.65 |
| HIC | EURO | Ireland | 1.15 | 14.2 | 4200000 | 139 | 3.31 | 0.58 |
| LIC | SEARO | Korea, Dem. People's Rep. | 0.00 | - | 25000000 | 120 | 0.48 | 0.50 |
| UMIC | EURO | Turkey | 0.96 | 15 | 63000000 | 108 | 0.17 | 0.45 |
| HIC | EURO | Czech Republic | 1.93 | 6.7 | 10000000 | 105 | 1.05 | 0.44 |
| UMIC | EURO | Hungary | 1.55 | 6.9 | 10000000 | 101 | 1.01 | 0.42 |
| HIC | EURO | Israel | 4.95 | - | 5100000 | 89 | 1.75 | 0.37 |
| HIC | WPRO | Korea, Rep. | 4.81 | - | 50000000 | 86 | 0.17 | 0.36 |
| LMIC | AFRO | Nigeria | 0.00 | - | 130000000 | 85 | 0.07 | 0.36 |
| HIC | EMRO | Saudi Arabia | 0.82 | 18.4 | 23000000 | 84 | 0.37 | 0.35 |
| UMIC | SEARO | Thailand | 1.00 | 5.1 | 64000000 | 84 | 0.13 | 0.35 |
| HIC | EURO | Austria | 3.17 | 9.3 | 8400000 | 83 | 0.99 | 0.35 |
| HIC | EURO | Estonia | 1.43 | 4.6 | 1300000 | 83 | 6.38 | 0.35 |
| HIC | EURO | Wales | 1.43 | - | 2900000 | 82 | 2.83 | 0.34 |
| HIC | EURO | Northern Ireland | 0.00 | - | 1700000 | 78 | 4.59 | 0.33 |
| UMIC | PAHO | Argentina | 0.54 | 18.2 | 36000000 | 74 | 0.21 | 0.31 |
| HIC | WPRO | Singapore | 1.94 | - | 4400000 | 71 | 1.61 | 0.30 |
| LMIC | WPRO | Vietnam | 0.53 | 4.1 | 87000000 | 67 | 0.08 | 0.28 |
| LIC | SEARO | Bangladesh | 0.07 | 1.3 | 150000000 | 61 | 0.04 | 0.26 |
| HIC | EURO | Croatia | 0.97 | 6.3 | 4300000 | 56 | 1.30 | 0.23 |
| HIC | EURO | Lithuania | 0.94 | 6 | 3300000 | 45 | 1.36 | 0.19 |
| LIC | AFRO | Ethiopia | 0.27 | 5.2 | 78000000 | 44 | 0.06 | 0.18 |
| HIC | EURO | Iceland | 2.03 | - | 284113 | 43 | 15.13 | 0.18 |
| UMIC | PAHO | Peru | 0.13 | - | 29000000 | 43 | 0.15 | 0.18 |
| LMIC | AFRO | Ghana | 0.00 | 4.7 | 24000000 | 41 | 0.17 | 0.17 |
| LMIC | EMRO | Pakistan | 0.24 | 10.8 | 170000000 | 41 | 0.02 | 0.17 |
| LMIC | AFRO | Kenya | 0.00 | 4.4 | 30000000 | 39 | 0.13 | 0.16 |
| HIC | PAHO | Puerto Rico | 0.43 | - | 3500000 | 39 | 1.11 | 0.16 |
| LMIC | SEARO | Sri Lanka | 0.11 | 6.9 | 21000000 | 39 | 0.19 | 0.16 |
| UMIC | PAHO | Ecuador | 0.44 | 11.4 | 15000000 | 37 | 0.25 | 0.16 |
| UMIC | PAHO | Costa Rica | 0.42 | - | 3900000 | 34 | 0.87 | 0.14 |
| LIC | SEARO | Nepal | 0.00 | 4.1 | 25000000 | 34 | 0.14 | 0.14 |
| HIC | EURO | Cyprus | 0.56 | 14.8 | 1100000 | 33 | 3.00 | 0.14 |
| LMIC | EURO | Georgia | 0.30 | 6 | 4000000 | 30 | 0.75 | 0.13 |
| UMIC | EMRO | Jordan | 0.71 | - | 6800000 | 30 | 0.44 | 0.13 |
| UMIC | PAHO | Venezuela, RB | 0.34 | - | 24000000 | 28 | 0.12 | 0.12 |
| HIC | EMRO | United Arab Emirates | 1.30 | 16.7 | 7000000 | 27 | 0.39 | 0.11 |
| UMIC | EURO | Bulgaria | 0.77 | 7.2 | 7800000 | 26 | 0.33 | 0.11 |
| LMIC | EMRO | Egypt, Arab Rep. | 0.72 | - | 65000000 | 26 | 0.04 | 0.11 |
| UMIC | EURO | Serbia | 0.92 | 18.2 | 7300000 | 26 | 0.36 | 0.11 |
| HIC | PAHO | Cuba | 0.43 | - | 11000000 | 25 | 0.23 | 0.10 |
| LIC | AFRO | Uganda | 0.17 | - | 33000000 | 25 | 0.08 | 0.10 |
| UMIC | EMRO | Lebanon | 0.00 | 12.5 | 4800000 | 24 | 0.50 | 0.10 |
| HIC | EMRO | Qatar | 0.51 | - | 1600000 | 24 | 1.50 | 0.10 |
| UMIC | EURO | Romania | 0.51 | 10.3 | 21000000 | 24 | 0.11 | 0.10 |
| HIC | EURO | Slovenia | 1.94 | 8 | 2000000 | 24 | 1.20 | 0.10 |
| LMIC | WPRO | Philippines | 0.16 | 6.3 | 85000000 | 23 | 0.03 | 0.10 |
| LMIC | SEARO | Indonesia | 0.23 | 8 | 240000000 | 22 | 0.01 | 0.09 |
| HIC | EURO | Russian Federation | 0.99 | 5.6 | 150000000 | 22 | 0.01 | 0.09 |
| HIC | EMRO | Oman | 0.22 | - | 3300000 | 21 | 0.64 | 0.09 |
| LMIC | AFRO | Cameroon | 0.00 | 10.9 | 21000000 | 20 | 0.10 | 0.08 |
| UMIC | PAHO | Jamaica | 0.00 | 12.8 | 2700000 | 20 | 0.74 | 0.08 |
| HIC | PAHO | Guatemala | 0.03 | 4.3 | 13000000 | 16 | 0.12 | 0.07 |
| HIC | EURO | Luxembourg | 1.24 | 12.7 | 513453 | 16 | 3.12 | 0.07 |
| LMIC | EMRO | Morocco | 0.00 | - | 32000000 | 16 | 0.05 | 0.07 |
| LMIC | EMRO | Palestine/West Bank and Gaza | 0.49 | - | 5790000 | 16 | 0.28 | 0.07 |
| UMIC | WPRO | Fiji | 0.00 | - | 791903 | 14 | 1.77 | 0.06 |
| UMIC | EMRO | Tunisia | 0.60 | 9.6 | 8400000 | 14 | 0.17 | 0.06 |
| LMIC | EURO | Ukraine | 0.47 | 4.9 | 46000000 | 14 | 0.03 | 0.06 |
| LIC | AFRO | Mozambique | 0.34 | 1.9 | 21000000 | 13 | 0.06 | 0.05 |
| LIC | AFRO | Tanzania | 0.51 | - | 45000000 | 13 | 0.03 | 0.05 |
| UMIC | EURO | Albania | 1.44 | - | 2900000 | 12 | 0.41 | 0.05 |
| HIC | EMRO | Bahrain | 0.10 | - | 947969 | 12 | 1.27 | 0.05 |
| HIC | EMRO | Kuwait | 0.06 | 17.2 | 3000000 | 12 | 0.40 | 0.05 |
| HIC | PAHO | Trinidad and Tobago | 0.09 | - | 1200000 | 12 | 1.00 | 0.05 |
| LMIC | WPRO | Vanuatu | 0.00 | - | 236237 | 11 | 4.66 | 0.05 |
| HIC | PAHO | Virgin Islands (U.S.) | 0.00 | - | 106734 | 11 | 10.31 | 0.05 |
| UMIC | EURO | Azerbaijan | 0.18 | - | 9600000 | 10 | 0.10 | 0.04 |
| HIC | PAHO | Barbados | 0.10 | 12.5 | 278852 | 10 | 3.59 | 0.04 |
| UMIC | AFRO | Botswana | 0.54 | 9.4 | 2200000 | 10 | 0.45 | 0.04 |
| LMIC | EURO | Kosovo | 0.00 | - | 1800000 | 10 | 0.56 | 0.04 |
| LMIC | WPRO | Mongolia | 0.10 | 2.5 | 2700000 | 10 | 0.37 | 0.04 |
| LMIC | AFRO | Senegal | 0.58 | 6.2 | 11000000 | 10 | 0.09 | 0.04 |
| HIC | EURO | Slovak Republic | 0.83 | 5.9 | 5400000 | 10 | 0.19 | 0.04 |
| HIC | PAHO | Uruguay | 0.48 | 9.1 | 3400000 | 10 | 0.29 | 0.04 |
| UMIC | WPRO | American Samoa | 2.16 | - | 55274 | 9 | 16.28 | 0.04 |
| HIC | WPRO | Guam | 0.00 | - | 161306 | 9 | 5.58 | 0.04 |
| HIC | EURO | Latvia | 0.63 | 8.5 | 2100000 | 9 | 0.43 | 0.04 |
| UMIC | EURO | Macedonia, FYR | 0.36 | - | 2100000 | 9 | 0.43 | 0.04 |
| LIC | SEARO | Myanmar | 0.03 | 3.4 | 53000000 | 9 | 0.02 | 0.04 |
| LMIC | PAHO | Paraguay | 0.15 | 11 | 6600000 | 9 | 0.14 | 0.04 |
| HIC | EURO | Greenland | 0.00 | - | 56216 | 8 | 14.23 | 0.03 |
| HIC | EURO | Malta | 0.57 | 19.2 | 407631 | 8 | 1.96 | 0.03 |
| UMIC | AFRO | Mauritius | 0.35 | 10.3 | 1200000 | 8 | 0.67 | 0.03 |
| LMIC | WPRO | Micronesia, Fed. Sts. | 0.00 | 17.7 | 106364 | 8 | 7.52 | 0.03 |
| LMIC | PAHO | Bolivia | 0.00 | - | 9100000 | 7 | 0.08 | 0.03 |
| LIC | AFRO | Malawi | 0.00 | 2.7 | 17000000 | 7 | 0.04 | 0.03 |
| LMIC | WPRO | Papua New Guinea | 0.03 | 5.2 | 6900000 | 7 | 0.10 | 0.03 |
| LMIC | WPRO | Samoa | 0.00 | 13.6 | 173441 | 7 | 4.04 | 0.03 |
| LIC | AFRO | Guinea | 0.00 | 3.2 | 11000000 | 6 | 0.05 | 0.03 |
| UMIC | PAHO | Suriname | 0.00 | - | 547253 | 6 | 1.10 | 0.03 |
| UMIC | WPRO | Tonga | 0.13 | 11.2 | 1100000 | 6 | 0.55 | 0.03 |
| LIC | AFRO | Burkina Faso | 0.70 | 4 | 18000000 | 5 | 0.03 | 0.02 |
| LIC | WPRO | Cambodia | 0.12 | 3 | 15000000 | 5 | 0.03 | 0.02 |
| HIC | WPRO | Northern Mariana Islands | 0.00 | - | 59403 | 5 | 8.42 | 0.02 |
| UMIC | AFRO | Algeria | 0.00 | 10.8 | 39000000 | 4 | 0.01 | 0.02 |
| LIC | AFRO | Benin | 0.00 | 2.4 | 9400000 | 4 | 0.04 | 0.02 |
| UMIC | EURO | Bosnia and Herzegovina | 0.20 | 9 | 3600000 | 4 | 0.11 | 0.02 |
| UMIC | PAHO | Dominican Republic | 0.00 | 16 | 10000000 | 4 | 0.04 | 0.02 |
| LMIC | PAHO | Honduras | 0.04 | - | 8400000 | 4 | 0.05 | 0.02 |
| UMIC | EMRO | Iraq | 0.04 | 15.6 | 35000000 | 4 | 0.01 | 0.02 |
| UMIC | EURO | Kazakhstan | 0.12 | 8.4 | 17000000 | 4 | 0.02 | 0.02 |
| LMIC | WPRO | Kiribati | 0.00 | 12.5 | 88906 | 4 | 4.50 | 0.02 |
| UMIC | EMRO | Libya | 0.00 | 12.2 | 6400000 | 4 | 0.06 | 0.02 |
| UMIC | AFRO | Seychelles | 0.22 | 6 | 92219 | 4 | 4.34 | 0.02 |
| LIC | EMRO | Somalia | 0.00 | - | 11000000 | 4 | 0.04 | 0.02 |
| LMIC | SEARO | Bhutan | 0.83 | 14 | 750888 | 3 | 0.40 | 0.01 |
| HIC | WPRO | Brunei Darussalam | 0.28 | - | 416284 | 3 | 0.72 | 0.01 |
| UMIC | WPRO | Marshall Islands | 0.00 | 13.2 | 53456 | 3 | 5.61 | 0.01 |
| HIC | WPRO | New Caledonia | 0.00 | - | 249675 | 3 | 1.20 | 0.01 |
| LMIC | PAHO | Nicaragua | 0.11 | - | 6100000 | 3 | 0.05 | 0.01 |
| UMIC | WPRO | Palau | 0.00 | - | 19796 | 3 | 15.15 | 0.01 |
| LMIC | EMRO | Sudan | 0.00 | - | 39000000 | 3 | 0.01 | 0.01 |
| LMIC | EMRO | Yemen, Rep. | 0.00 | - | 20000000 | 3 | 0.02 | 0.01 |
| LMIC | EURO | Armenia | 0.19 | - | 3000000 | 2 | 0.07 | 0.01 |
| LIC | AFRO | Comoros | 0.00 | 2.2 | 693957 | 2 | 0.29 | 0.01 |
| LMIC | AFRO | Côte d'Ivoire | 0.10 | 8.8 | 23000000 | 2 | 0.01 | 0.01 |
| LIC | AFRO | Gambia, The | 0.07 | 6.5 | 890528 | 2 | 0.22 | 0.01 |
| LMIC | PAHO | Guyana | 0.00 | - | 766487 | 2 | 0.26 | 0.01 |
| LIC | PAHO | Haiti | 0.00 | - | 11000000 | 2 | 0.02 | 0.01 |
| LMIC | EURO | Kyrgyz Republic | 0.11 | - | 5900000 | 2 | 0.03 | 0.01 |
| HIC | WPRO | Macao SAR, China | 0.20 | - | 588135 | 2 | 0.34 | 0.01 |
| LMIC | AFRO | Mauritania | 0.01 | 11.7 | 3600000 | 2 | 0.06 | 0.01 |
| UMIC | EURO | Montenegro | 0.37 | - | 621595 | 2 | 0.32 | 0.01 |
| UMIC | PAHO | Panama | 0.15 | - | 3300000 | 2 | 0.06 | 0.01 |
| LIC | AFRO | Rwanda | 0.67 | - | 11000000 | 2 | 0.02 | 0.01 |
| LMIC | WPRO | Solomon Islands | 0.00 | 11.7 | 472059 | 2 | 0.42 | 0.01 |
| LMIC | EMRO | Syrian Arab Republic | 0.02 | - | 19000000 | 2 | 0.01 | 0.01 |
| LMIC | SEARO | Timor-Leste | 0.00 | - | 1200000 | 2 | 0.17 | 0.01 |
| LIC | AFRO | Zimbabwe | 0.00 | 6.4 | 15000000 | 2 | 0.01 | 0.01 |
| UMIC | PAHO | Belize | 0.00 | - | 356041 | 1 | 0.28 | 0.00 |
| LMIC | AFRO | Cabo Verde | 0.00 | 5.5 | 518763 | 1 | 0.19 | 0.00 |
| LIC | AFRO | Chad | 0.30 | 6.5 | 14000000 | 1 | 0.01 | 0.00 |
| HIC | PAHO | Curaçao | 0.00 | - | 144495 | 1 | 0.69 | 0.00 |
| LMIC | PAHO | El Salvador | 0.18 | - | 6200000 | 1 | 0.02 | 0.00 |
| UMIC | PAHO | Grenada | 0.00 | - | 107776 | 1 | 0.93 | 0.00 |
| LIC | AFRO | Mali | 0.29 | 5.6 | 17000000 | 1 | 0.01 | 0.00 |
| UMIC | AFRO | Namibia | 0.34 | 15.6 | 2400000 | 1 | 0.04 | 0.00 |
| LIC | AFRO | Niger | 0.00 | 7.8 | 20000000 | 1 | 0.01 | 0.00 |
| UMIC | PAHO | St. Lucia | 0.00 | - | 181748 | 1 | 0.55 | 0.00 |
| LMIC | AFRO | Swaziland | 0.00 | 18.4 | 1100000 | 1 | 0.09 | 0.00 |
| LIC | AFRO | Togo | 0.27 | - | 7200000 | 1 | 0.01 | 0.00 |
| HIC | PAHO | Turks and Caicos Islands | 0.00 | - | 34585 | 1 | 2.89 | 0.00 |
| LMIC | EURO | Uzbekistan | 0.16 | - | 26000000 | 1 | 0.00 | 0.00 |
| LMIC | AFRO | Zambia | 0.00 | 4.6 | 16000000 | 1 | 0.01 | 0.00 |
| LIC | EMRO | Afghanistan | 0.00 | - | 15913526 | 0 | 0.00 | 0.00 |
| HIC | EURO | Andorra | 0.00 | - | 61414 | 0 | 0.00 | 0.00 |
| UMIC | AFRO | Angola | 0.03 | - | 12840332 | 0 | 0.00 | 0.00 |
| HIC | PAHO | Antigua and Barbuda | 0.00 | - | 68586 | 0 | 0.00 | 0.00 |
| HIC | PAHO | Aruba | 0.00 | - | 76945 | 0 | 0.00 | 0.00 |
| HIC | PAHO | Bahamas, The | 0.00 | - | 276519 | 0 | 0.00 | 0.00 |
| UMIC | EURO | Belarus | 0.61 | - | 9481386 | 0 | 0.00 | 0.00 |
| HIC | PAHO | Bermuda | 0.26 | - | 59777 | 0 | 0.00 | 0.00 |
| LIC | AFRO | Burundi | 0.21 | - | 6155011 | 0 | 0.00 | 0.00 |
| HIC | PAHO | Cayman Islands | 0.00 | - | 31568 | 0 | 0.00 | 0.00 |
| LIC | AFRO | Central African Republic | 0.00 | - | 3294372 | 0 | 0.00 | 0.00 |
| HIC | EURO | Channel Islands | 0.00 | - | 143965 | 0 | 0.00 | 0.00 |
| LIC | AFRO | Congo, Dem. Rep | 0.41 | 12.1 | 41177784 | 0 | 0.00 | 0.00 |
| LMIC | AFRO | Congo Rep. | 0.00 | 13 | 2695857 | 0 | 0.00 | 0.00 |
| LMIC | EMRO | Djibouti | 0.00 | - | 654402 | 0 | 0.00 | 0.00 |
| UMIC | PAHO | Dominica | 0.00 | 6.5 | 71813 | 0 | 0.00 | 0.00 |
| HIC | AFRO | Equatorial Guinea | 0.00 | - | 442512 | 0 | 0.00 | 0.00 |
| LIC | AFRO | Eritrea | 0.00 | 10.8 | 3215321 | 0 | 0.00 | 0.00 |
| HIC | EURO | Faeroe Islands | 0.00 | - | 45600 | 0 | 0.00 | 0.00 |
| HIC | WPRO | French Polynesia | 0.00 | - | 214514 | 0 | 0.00 | 0.00 |
| UMIC | AFRO | Gabon | 0.00 | 9.8 | 1074579 | 0 | 0.00 | 0.00 |
| LIC | AFRO | Guinea-Bissau | 0.00 | - | 1169548 | 0 | 0.00 | 0.00 |
| HIC | EURO | Isle of Man | 0.00 | - | 72542 | 0 | 0.00 | 0.00 |
| LMIC | WPRO | Lao PDR | 0.00 | 5 | 4779474 | 0 | 0.00 | 0.00 |
| LMIC | AFRO | Lesotho | 0.05 | - | 1730035 | 0 | 0.00 | 0.00 |
| LIC | AFRO | Liberia | 0.00 | - | 2216596 | 0 | 0.00 | 0.00 |
| HIC | EURO | Liechtenstein | 0.00 | - | 30685 | 0 | 0.00 | 0.00 |
| LIC | AFRO | Madagascar | 0.01 | - | 13310904 | 0 | 0.00 | 0.00 |
| UMIC | SEARO | Maldives | 0.00 | 10.4 | 249800 | 0 | 0.00 | 0.00 |
| LMIC | EURO | Moldova | 0.25 | - | 3547100 | 0 | 0.00 | 0.00 |
| HIC | EURO | Monaco | 0.00 | - | 30571 | 0 | 0.00 | 0.00 |
| HIC | EURO | San Marino | 0.00 | - | 25632 | 0 | 0.00 | 0.00 |
| LMIC | AFRO | Sao Tome and Principe | 0.00 | 5.1 | 124262 | 0 | 0.00 | 0.00 |
| LIC | AFRO | Sierra Leone | 0.00 | 5.3 | 3890035 | 0 | 0.00 | 0.00 |
| HIC | PAHO | Sint Maarten (Dutch part) | 0.00 | - | 35937 | 0 | 0.00 | 0.00 |
| LMIC | EMRO | South Sudan | 0.00 | - | 5729906 | 0 | 0.00 | 0.00 |
| HIC | PAHO | St. Kitts and Nevis | 0.00 | 10.2 | 48703 | 0 | 0.00 | 0.00 |
| HIC | PAHO | St. Martin (French part) | 0.00 | - | 28291 | 0 | 0.00 | 0.00 |
| UMIC | PAHO | St. Vincent and the Grenadines | 0.00 | - | 107955 | 0 | 0.00 | 0.00 |
| LIC | EURO | Tajikistan | 0.10 | - | 5724722 | 0 | 0.00 | 0.00 |
| UMIC | EURO | Turkmenistan | 0.00 | - | 4104616 | 0 | 0.00 | 0.00 |
| UMIC | WPRO | Tuvalu | 0.00 | - | 9200 | 0 | 0.00 | 0.00 |
| ***World Health Organization regions: Africa - AFRO; Eastern Mediterranean EMRO; Europe - EURO; The Americas and the Caribbean - PAHO; South East Asia - SEARO; Western Pacific - WPRO | | | | | | | | |
| ****World Bank income classification: High Income - HIC; Upper middle income - UMIC; Lower middle income - LMIC; Low income - LIC | | | | | | | | |

# Appendix Figure 1. Publication rate per 100.000 inhabitants by decade of publication by study design


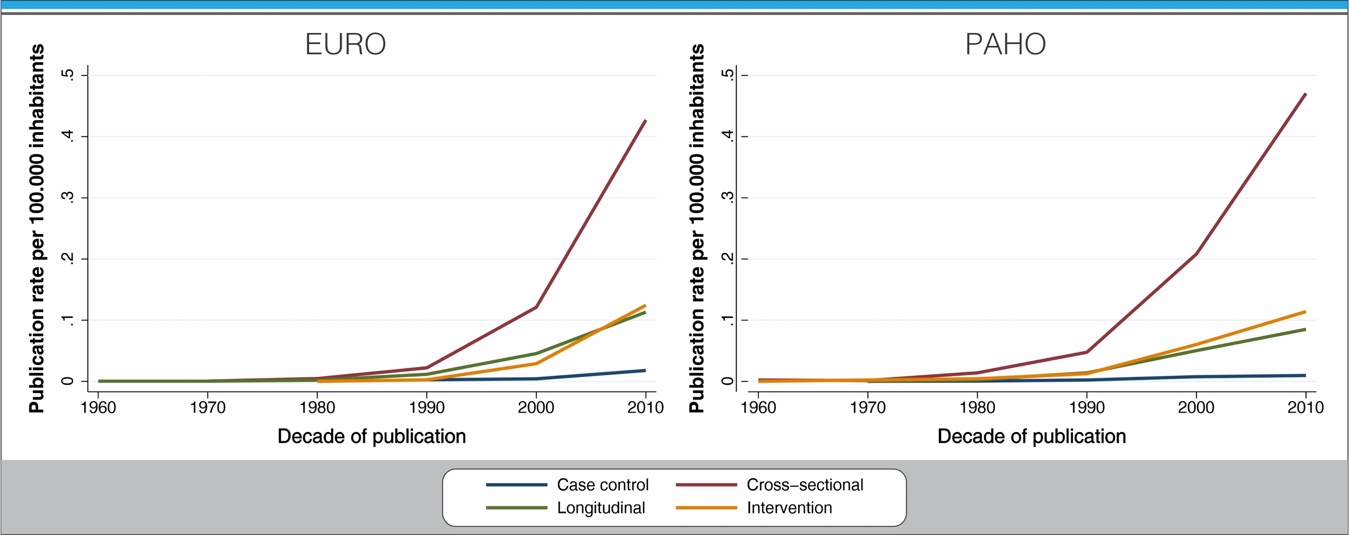


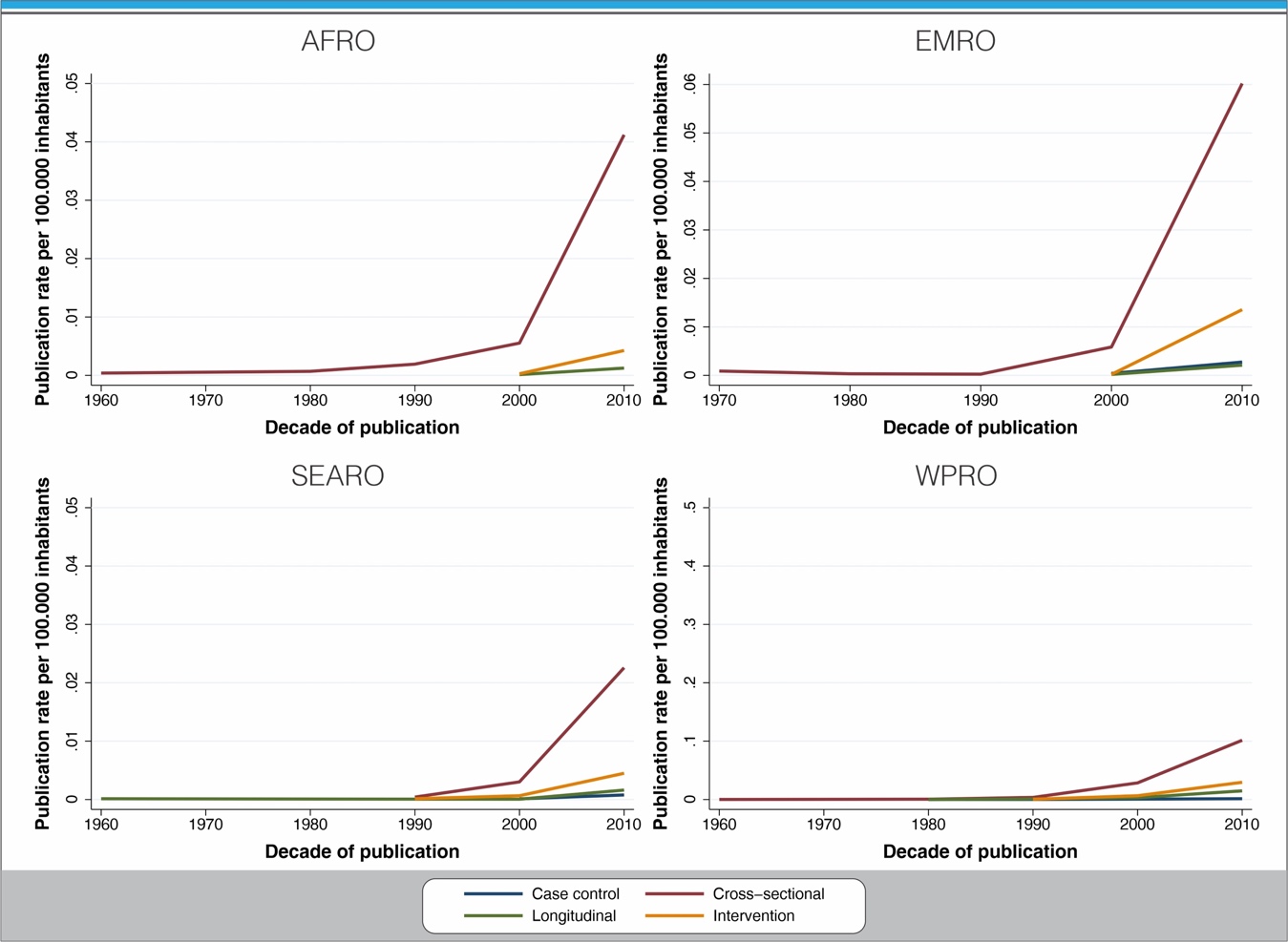


# Appendix Figure 2. Publication rate per 100,000 inhabitants by decade of publication by study`s population age group by world regions


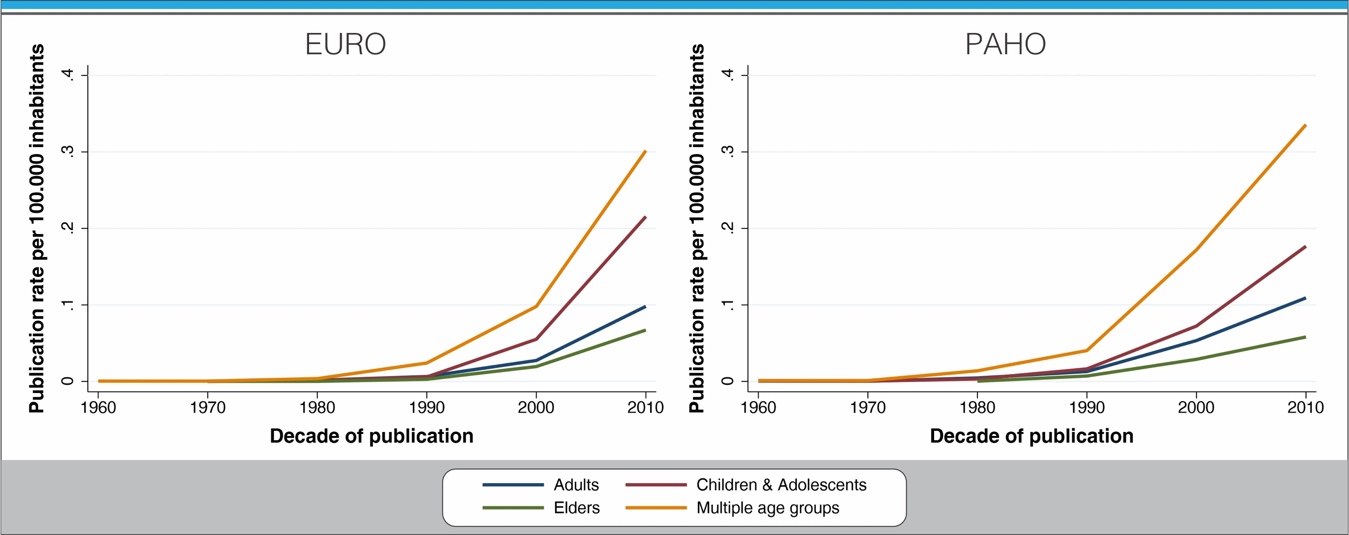


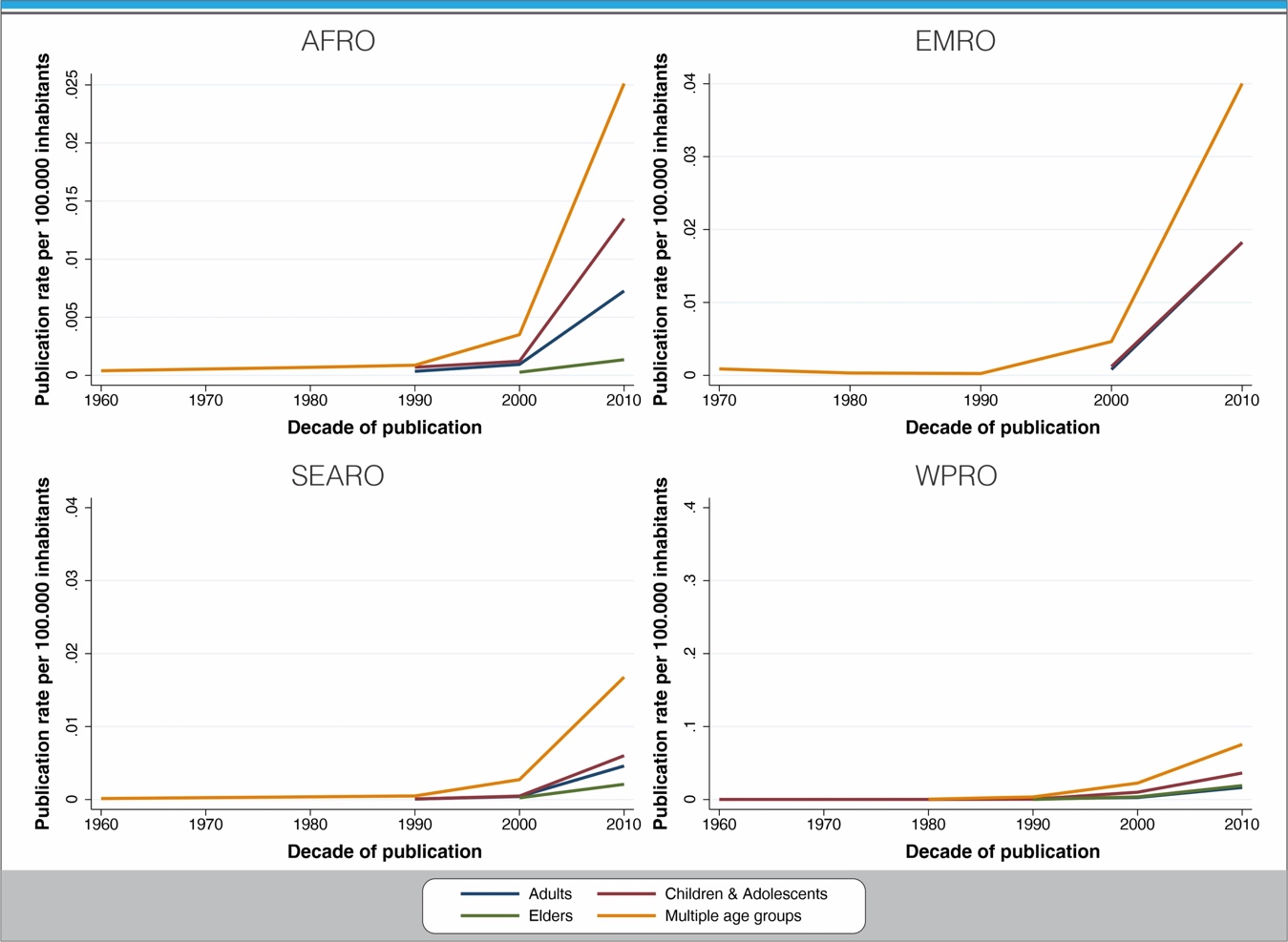


# Appendix Figure 3. Worldwide time trends in physical activity research by income group, 1950-2019


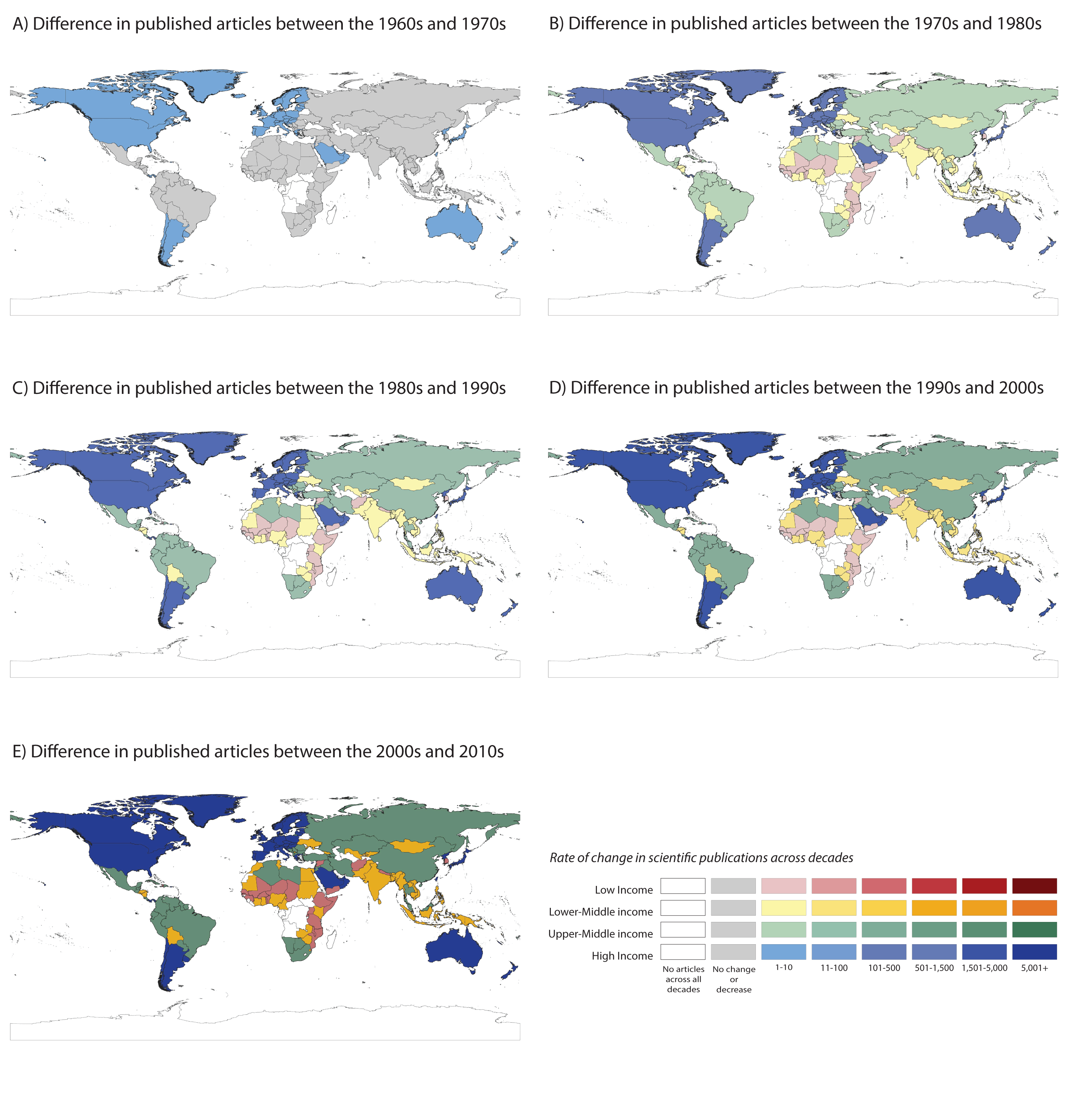


# Appendix figure 4. Worldwide time trends in physical activity prevalence measurement and trends research, 1950-2019


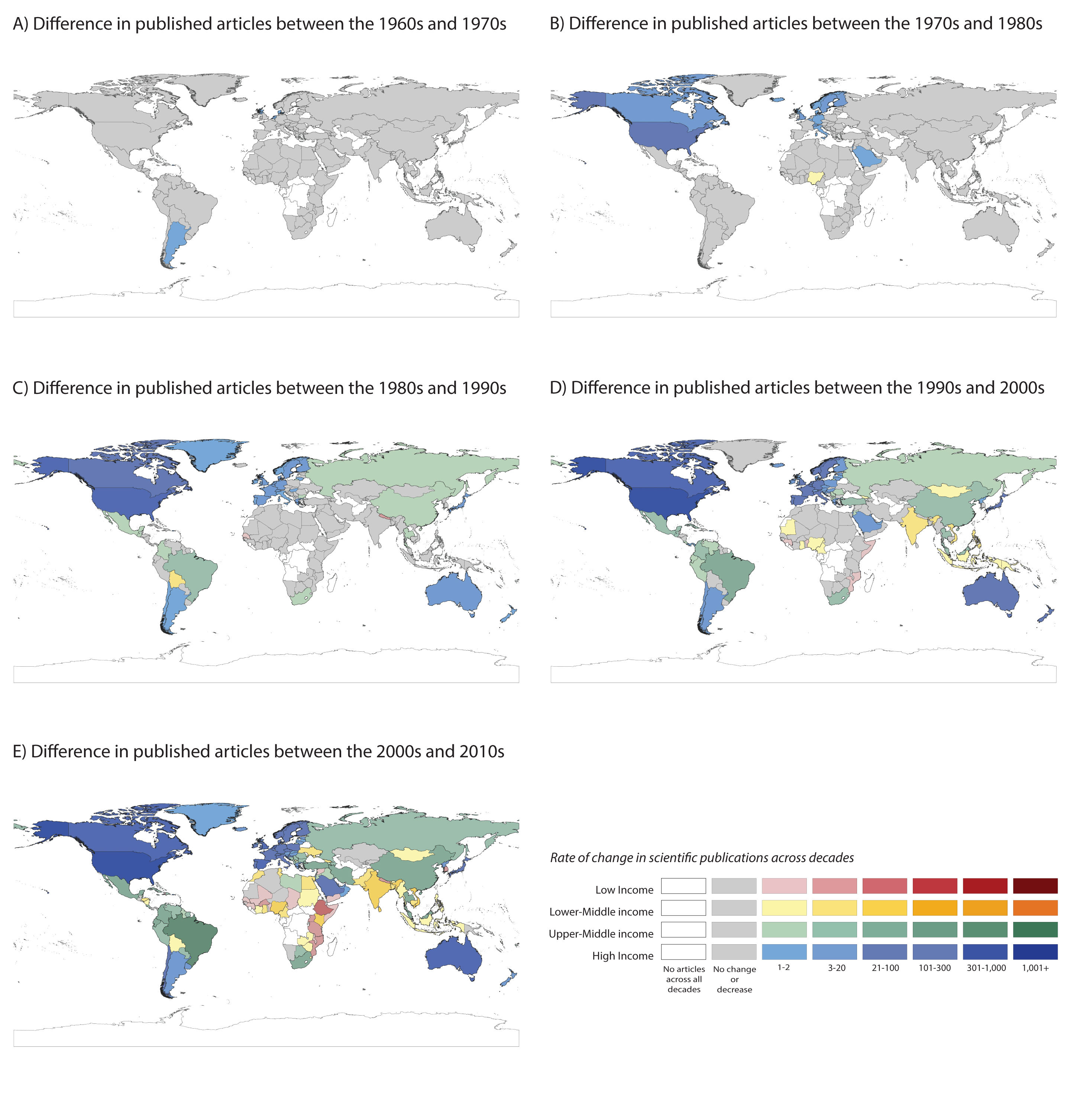


# Appendix figure 5. Worldwide time trends in physical activity correlates and determinants research, 1950-2019


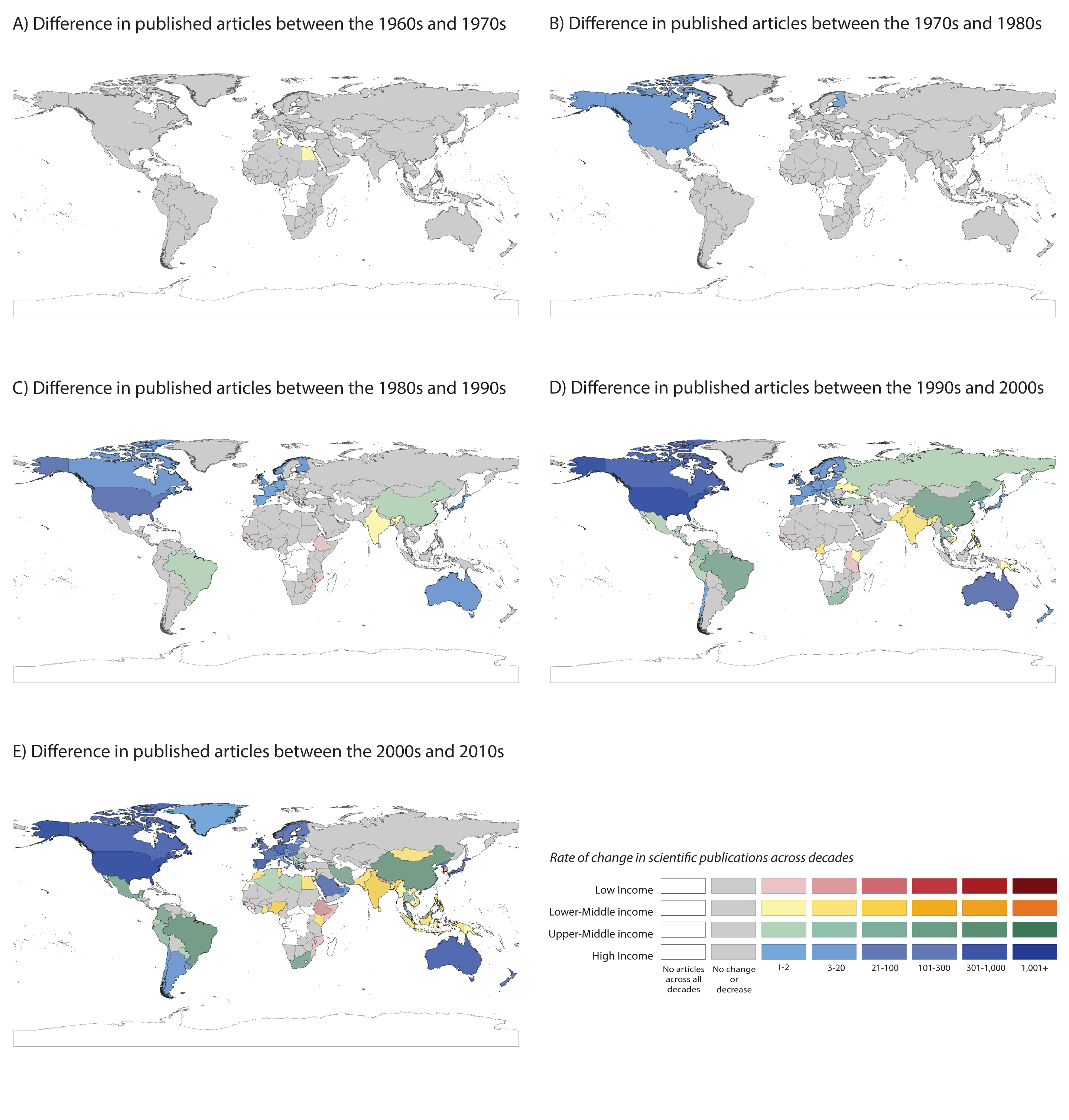


# Appendix figure 6. Worldwide time trends in physical activity health consequences research, 1950-2019


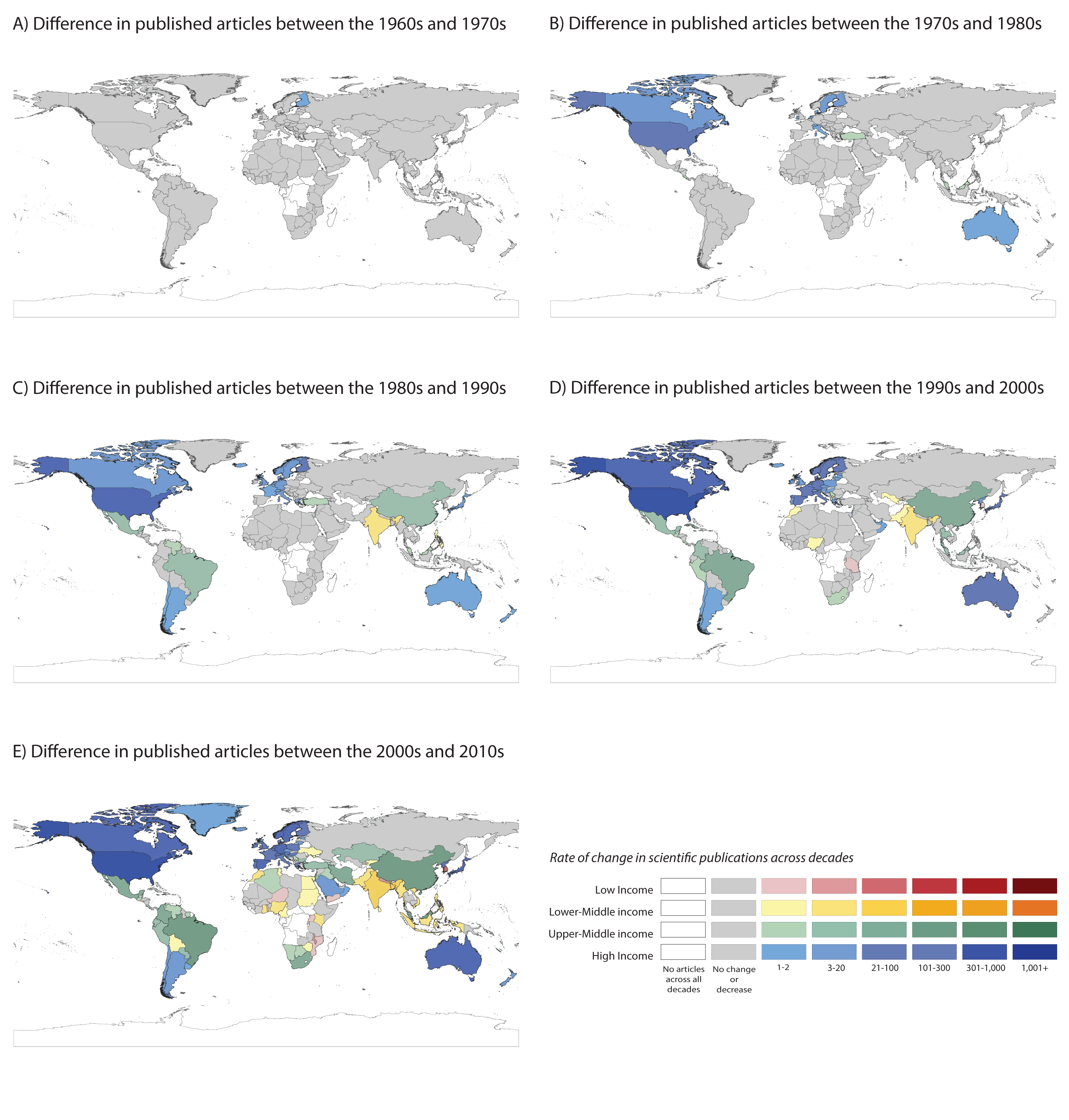


# Appendix figure 7. Worldwide time trends in physical activity intervention research, 1950-2019


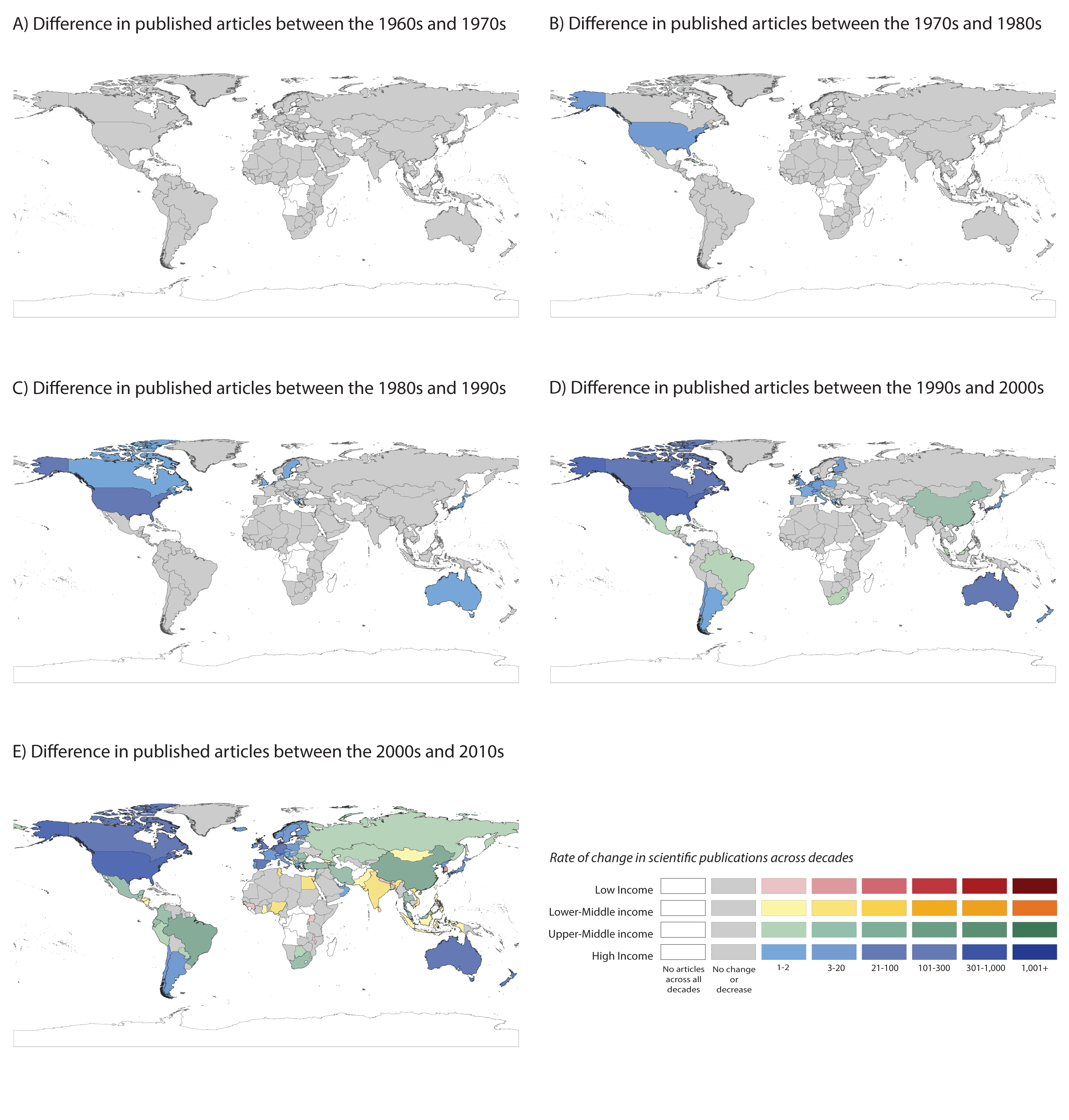


# Appendix figure 8. Worldwide time trends in physical activity policy research, 1950-2019


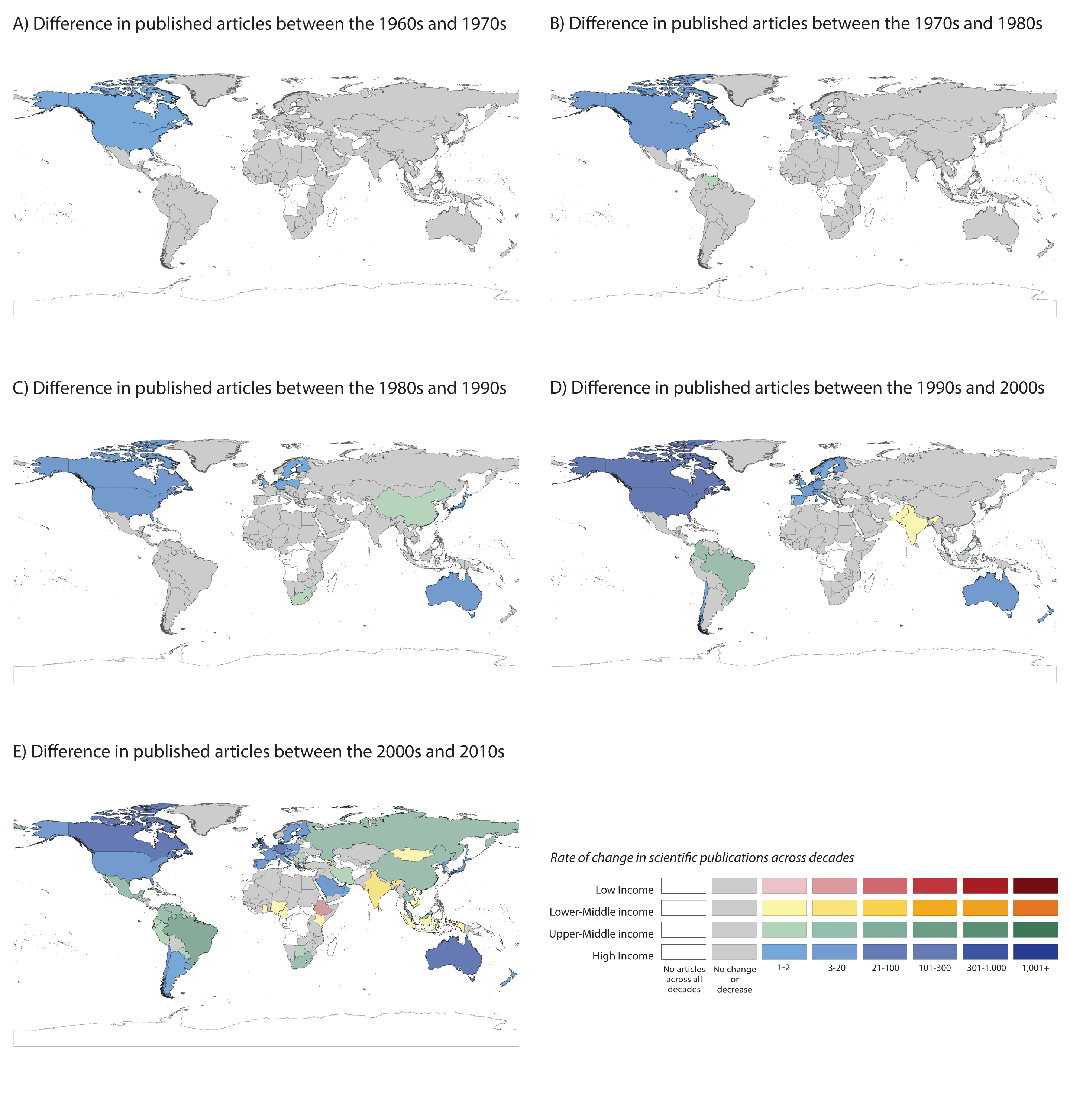


# Appendix figure 9. Worldwide research productivity of fields related to physical activity since 1950


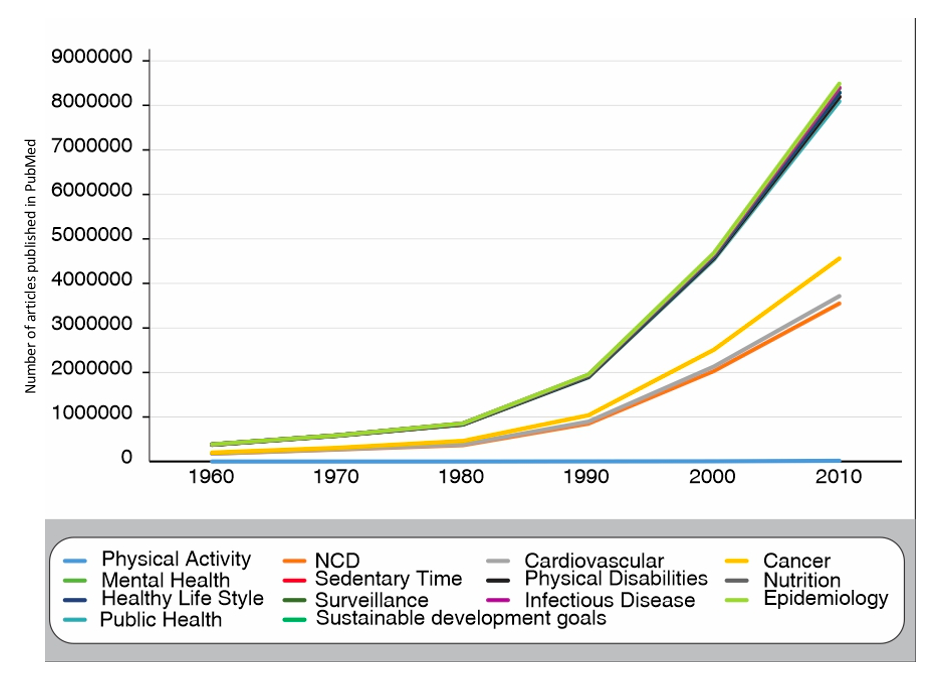

Supplement: Supplementary file 1 — Additional file 1: Supplementary appendix. Webtable 1. Physical activity research characteristics per country, 1950–2019. Appendix Figure 1. Publication rate per 100.000 inhabitants by decade of publication by study design. Appendix Figure 2. Publication rate per 100,000 inhabitants by decade of publication by study’s population age group by world regions. Appendix Figure 3. Worldwide time trends in physical activity research by income group, 1950–2019. Appendix Figure 4. Worldwide time trends in physical activity prevalence measurement and trends research, 1950–2019. Appendix Figure 5. Worldwide time trends in physical activity correlates and determinants research, 1950–2019. Appendix Figure 6. Worldwide time trends in physical activity health consequences research, 1950–2019. Appendix Figure 7. Worldwide time trends in physical activity intervention research, 1950–2019. Appendix Figure 8. Worldwide time trends in physical activity policy research, 1950–2019. Appendix Figure 9. Worldwide research productivity of fields related to physical activity since 1950. [file 12966_2020_1071_MOESM1_ESM.docx]
